# Analysis of the structural diversity of heterocycles amongst European medicines agency approved pharmaceuticals (2014–2023)

**DOI:** 10.1039/d5md00403a

**Published:** 2025-08-11

**Authors:** Matthew Ward, Niamh M. O'Boyle

**Affiliations:** a School of Pharmacy and Pharmaceutical Sciences, Trinity Biomedical Sciences Institute, Trinity College Dublin 152 – 160 Pearse St Dublin 2 D02 R590 Ireland nioboyle@tcd.ie; b Viatris Damastown, Damastown Industrial Park Damastown Road, Damastown Dublin D15 XD71 Ireland

## Abstract

This review presents a detailed analysis of the heterocycle diversity amongst medicines with new active substances (NAS) approved by the European Medicines Agency (EMA) in the 10 years from 2014–2023. A total of 380 medicines were approved that contain a NAS, of which 160 are small molecule products that contained one or more NAS with a heterocycle (164 NAS in total). Of the 164 heterocycle-containing NAS, 76% contained more than one heterocycle. The majority (59%) of the 164 active substances contained at least one fused heterocycle. The most common bicyclic rings were quinoline, benzimidazole, indole, and pyrrolopyrimidine. Tricyclic and polycyclic fused rings were observed but were rare. There were 28 distinct monocyclic heterocycles, consisting of 3, 4, 5, and 6 membered rings. 5-Membered rings were the most diverse as 15 of the 28 heterocycles are 5-membered rings. 6-Membered rings ranked second with 12 heterocycles. There was one 3-membered ring and one 4-membered ring seen. Nitrogen was by far the most common heteroatom in both monocyclic and fused heterocycles. Oxygen, sulfur and boron appeared in monocyclic heterocycles, whilst oxygen, sulfur and phosphorous were noted in fused heterocycles. The most common monocyclic heterocycles were pyridine, piperidine, pyrrolidine, piperazine, pyrimidine, pyrazole, triazole, imidazole and tetrahydropyran. This analysis provides valuable information on the structural diversity of heterocycles that were present in EMA approved medicines between 2014–2023. It highlights heterocycle occurrences, diversity, substitution patterns, and trends. The information detailed will be of interest to organic chemists, researchers, regulatory agencies, and the pharmaceutical industry as it demonstrates how common heterocycles are seen amongst EMA approved medicines for a wide range of therapeutic areas.

## Abstract 2

This review presents a detailed analysis of the heterocycle diversity amongst medicines with small molecule new active substances (NAS) approved by the European Medicines Agency (EMA) in the decade from 2014–2023.

## Graphical abstract

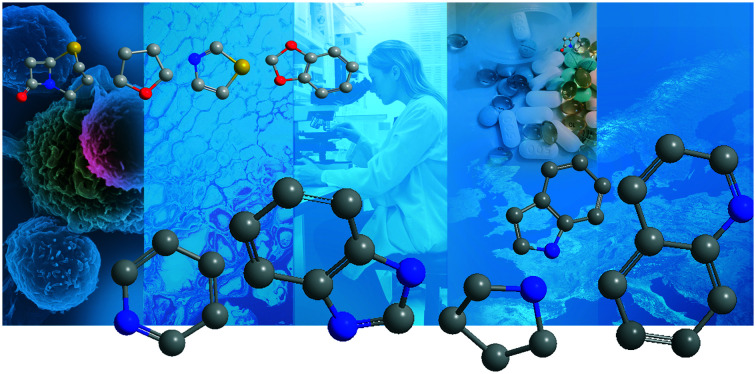

## Introduction

Heterocyclic compounds, also known as heterocycles, are cyclic compounds in which the ring members contain atoms of at least two different elements.^1^ Heterocycles are regarded as one of the most prominent and diverse class of compounds.^2^ They are amongst the largest and most varied families of organic compounds. The reason for such variety may be explained by the relatively simple requirements that must be met for classification as a heterocycle. The enormous degree of structural variety amongst heterocycles can be traced back to different factors including number of atoms in the ring, ring saturation or unsaturation, the type of heteroatoms in the ring, presence of multiple heteroatoms in the ring, structural isomers, and if the ring is fused or shares its atoms with another ring. Inorganic heterocycles, *e.g.* bicyclo[3.3.1]tetrasiloxane, are less common. The most frequently reported non-carbon atoms in heterocycles are nitrogen, oxygen, and sulfur.^3^ It has been estimated that nitrogen heterocycles are present in 59–82% of small molecule drugs.^4,5^

Due to their frequent presence within drug active substances, there is substantial interest in heterocycles. As heterocycles are so common across all drugs, they are seen in medicines that treat a wide range of therapeutic areas. The curiosity in heterocycle containing compounds is because they are not limited to one or two diseases or disorders, and they are present in treatments for a range of diseases such as cancer, infections, hormone disorders, and cardiovascular diseases.^3^

Although heterocycles are one of the most important classes of compounds found across all drugs, there are no studies that look specifically at the heterocycles found amongst drugs that have been approved for use in Europe by the European Medicines Agency (EMA). Previous work analysed the structural diversity, substitution patterns and frequency of nitrogen heterocycles within US FDA approved drugs between 1938–2012 and 2013–2023.^4,5^ Due to the lack of information about structural diversity of EMA approvals, this perspective will analyse all types of heterocycles in drugs approved by the EMA in the decade between January 2014 – December 2023.

## Methods

Details of medicines approved centrally by the EMA are publicly available from the EMA website. All centrally approved medicines are included in this analysis. The EMA centralised procedure is compulsory for medicines containing a new active substance to treat human immunodeficiency virus (HIV) or acquired immune deficiency syndrome (AIDS), cancer, diabetes, neurodegenerative diseases, auto-immune and other immune dysfunctions and viral diseases, medicines derived from biotechnology processes, advanced-therapy medicines, and orphan medicines. Other medicines may be authorised in individual European Union Member States *via* national procedures, rather than centrally *via* the EMA, and these medicines are not included in the analysis.

### Search strategy for EMA approved drugs between 2015–2023

A data collection method similar to previous work^6^ was used over the 10-year period (2014–2023). This approach focuses on medicines that are described as “new active substances (NAS)”. NAS are defined as “*chemical, biological or radiopharmaceutical substances not previously authorised in a medicinal product for human use in the European Union*”.^7^ From 2015 onwards, the EMA published reports titled “*Human Medicines: Highlights of (year)*”. These reports highlight the NAS that were recommended for approval in that year. An evaluation of the EMA reports from 2015–2023 was performed. From this a list was created that contained the proprietary names of all EMA medicines recommended for approval that were classified as NAS. However, the date of recommendation and date of marketing authorisation can be in different years. In this analysis, the year that the marketing authorisation was issued was considered to be the date of approval. For example, Omjjara was recommended for approval by the EMA in 2023, but the marketing authorisation was not issued until 2024. Hence, it is not included in this analysis. The marketing authorisation date for each NAS was examined and this provided the list of EMA approved drugs between 2015–2023.

### Drugs approved by the EMA in 2014

A different approach was utilised for 2014 as this was before the EMA began publishing yearly highlights. The “*European Public Assessment Reports (EPAR)*” was reviewed for medicines approved in 2014. The EPAR is a central database that contains all medicines authorised in the EU. The database was filtered for 2014 only and generics and biosimilars were removed using the built-in filters on the EPAR report. In the case of NAS that appear in multiple medicines, they were counted once (*i.e.* on their first approval as a NAS). For example, the NAS sofosbuvir appeared in two medicines (Sovaldi and Harvoni) approved in 2014 but was counted once.

The proprietary names for the drugs and therapeutic areas for 2014 were added to the data for 2015–2023 to capture all NAS across the 10-year period (2014–2023). Of the 160 medicines approved, 11 were later withdrawn and one (Oxbryta) had its marketing authorisation suspended. Although these drugs were withdrawn or suspended, they were approved by the EMA in the review period and were included in this evaluation.

### Obtaining NAS within all EMA approved medicines in the 10-year period

The list created above contains the proprietary names for medicines with a NAS approval between 2014–2023 and was then cross-referenced against the EMA website to extract the details of the NAS. The NAS in each medicine were extracted from the European Public Assessment Reports (EPAR). The medicines proprietary names were now correlated to the NAS they contain.

For combination products containing more than one active substance, only NAS were considered in this analysis. For example, vabomere contains the two active substances meropenem and vaborbactam. As meropenem was previously approved prior to 2014, it was not included in this analysis.

### Exclusion criteria for analysis

The scope of this EMA review was strictly new chemical substances containing at least one heterocycle, *i.e.* those that are covered by ICH Q6A.^8^ In addition, two low molecular weight radiopharmaceuticals [piflufolastat (18F) and flutemetamol (18F)] were included; other radiopharmaceuticals were excluded due to their classification as peptides or lack of heterocycle in their structure. The list of NAS was filtered to remove all biopharmaceuticals (vaccines, enzymes, biologics, monoclonal antibodies (mAbs), cell and gene therapies, peptides and glycopeptides), some of which are covered under ICH Q6B specifications (test procedures and acceptance criteria for biotechnological/biological products)^9^ or advanced therapy medicinal products (ATMP) guidelines.^10^ The World Health Organisation (WHO) guidance on the use of international non-proprietary names for pharmaceutical substances was used to identify biologic drugs using common stems. Any NAS that contained the following suffixes was removed: -mab (monoclonal antibody), -ase (enzymes), -gene (gene therapy products), – tide (peptides and glycopeptides). Categories of drugs excluded from the analysis are available in SI Table S1.

### Extracting and examining NAS that contain a heterocycle within the filtered medicines

The chemical structures of each NAS were extracted from online databases (Drugbank and PubChem). In the initial search, the structure was classified as heterocycle-containing or not. The final part of the EMA breakdown involved identifying medicines that contained more than one NAS with a heterocycle.

The list created now only contained medicines with at least one heterocycle in their NAS(s). The heterocycle was classified based on the IUPAC name of the NAS. The heterocycles within the relevant NAS were classified according to the following categories:

• Ring type (number of atoms in the ring).

• Number of heterocycles within each NAS.

• Types and number of heteroatoms in the ring.

• Saturated and unsaturated heterocycles.

• Fused and non-fused heterocycles. Fused and non-fused heterocycles were classified separately. For example, ledispavir was classified as containing an imidazole ring, whereas daridorexant was classified as containing a benzimidazole ring. Of note, isothiazole, oxaborolane, dioxolane, thiazine, triazinane, trioxane and dioxaphosphinane were only present as fused rings. All instances of 7-membered rings were in fused configurations only. Fused heterocycles were further classified as bicyclic, tricyclic or polycyclic. Spiro compounds ledispavir, rolapitant, apalutamide, atogepant and risdiplam, were uncommon.

• Bridged bicyclic compounds and inorganic rings.

## Results and discussion

Prior to examining the structural diversity amongst the heterocycles in EMA approved drugs, the therapeutic areas for all 380 approvals in the review period were analysed (Fig. S1). Anticancer medicines were the dominant area for approvals (27%), followed by neurology. There were 43 medicines approved to treat infections (11%), 40 medicines for blood disorders (11%), 36 medicines for neurology (9%), and 25 medicines for endocrinology (7%).

### Overview of heterocycles in EMA approved small molecule drugs (2014–2023)

**Fig. 1:**
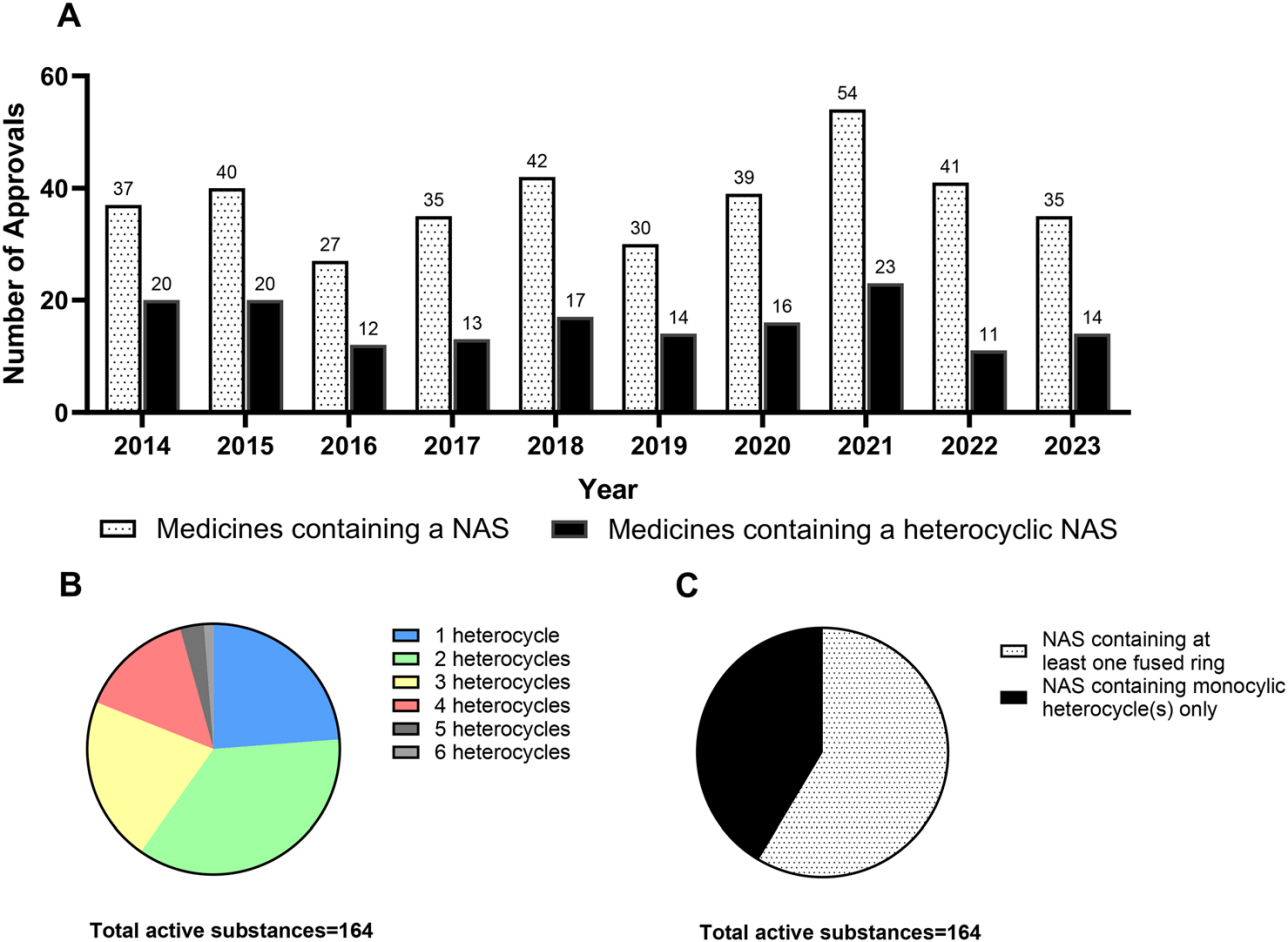
A. Breakdown of medicines containing a NAS and heterocyclic NAS in EMA approved medicines (2014–2023). Note that some medicines contain more than one NAS. B. Number of heterocycles per NAS; C. Proportion of NAS containing at least one fused heterocycle compared to NAS containing only monocyclic heterocycle(s).

There were 380 NAS approvals by the EMA between 2014–2023 (including biologicals, *etc.*). Of the 380 approved medicines, 160 were small molecules containing at least one heterocyclic NAS (Fig. 1A). Within the 160 products there were a total of 164 unique NAS that contain a heterocycle, as the medicines Maviret, Lonsurf, Zepatier, and Harvoni are combination products with two NAS containing heterocycles.

**Fig. 2:**
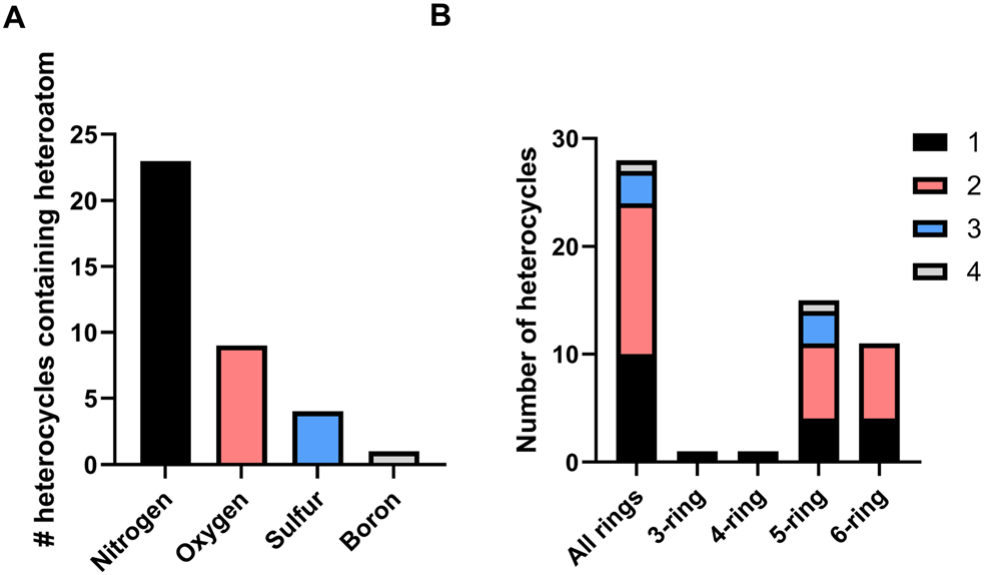
Analysis of monocyclic heterocycles in EMA drug approvals 2014–2023. A. Number of heterocycles containing different heteroatoms. Note that a heterocycle may contain more than one heteroatom; B. number of heteroatoms in each ring by total number of rings and per size of ring.

The number of heterocycles in each of the 164 NAS was also analysed (Fig. 2B). The most popular class was those with two heterocycles per NAS (36%), followed by one heterocycle per NAS (24%) and three heterocycles per NAS (21%). Five NAS contained five heterocycles (*e.g.* avatrombopag) and two NAS contained six heterocycles (*e.g.* pibrentasvir). Of the 164 NAS, 96 NAS (59%) contained at least one fused heterocycle, and 68 NAS (41%) contained only a monocyclic heterocycle(s) (Fig. 1C). There were 28 different monocyclic heterocycles identified, alongside 35 bicyclic heterocycles, 12 tricyclic heterocycles, four polycyclic heterocycles, eight bridged heterocyclic compounds and one inorganic heterocycle.

### Monocyclic heterocycles within EMA approved drugs (2014–2023)

There were 28 different monocyclic heterocycles identified. Each NAS was classified according to the heteroatom(s) in the ring, and the number of heteroatoms in their ring. Only four non-carbon atoms were present in the monocyclic heterocycles (Fig. 2A). Nitrogen was the most common heteroatom occurring in 82% of all monocyclic heterocycles, followed by oxygen (32%) and sulfur (14%). Boron was the only other heteroatom observed, appearing just once in the beta-lactamase inhibitor vaborbactam. The majority of heterocycles contained more than one heteroatom in their structure (Fig. 2B). 50% of heterocycles contained two heteroatoms, with 36% of heterocycles containing one heteroatom and 11% of heterocycles containing three heteroatoms.

### Three- and four-membered heterocycles amongst EMA approved drugs

**Fig. 3:**
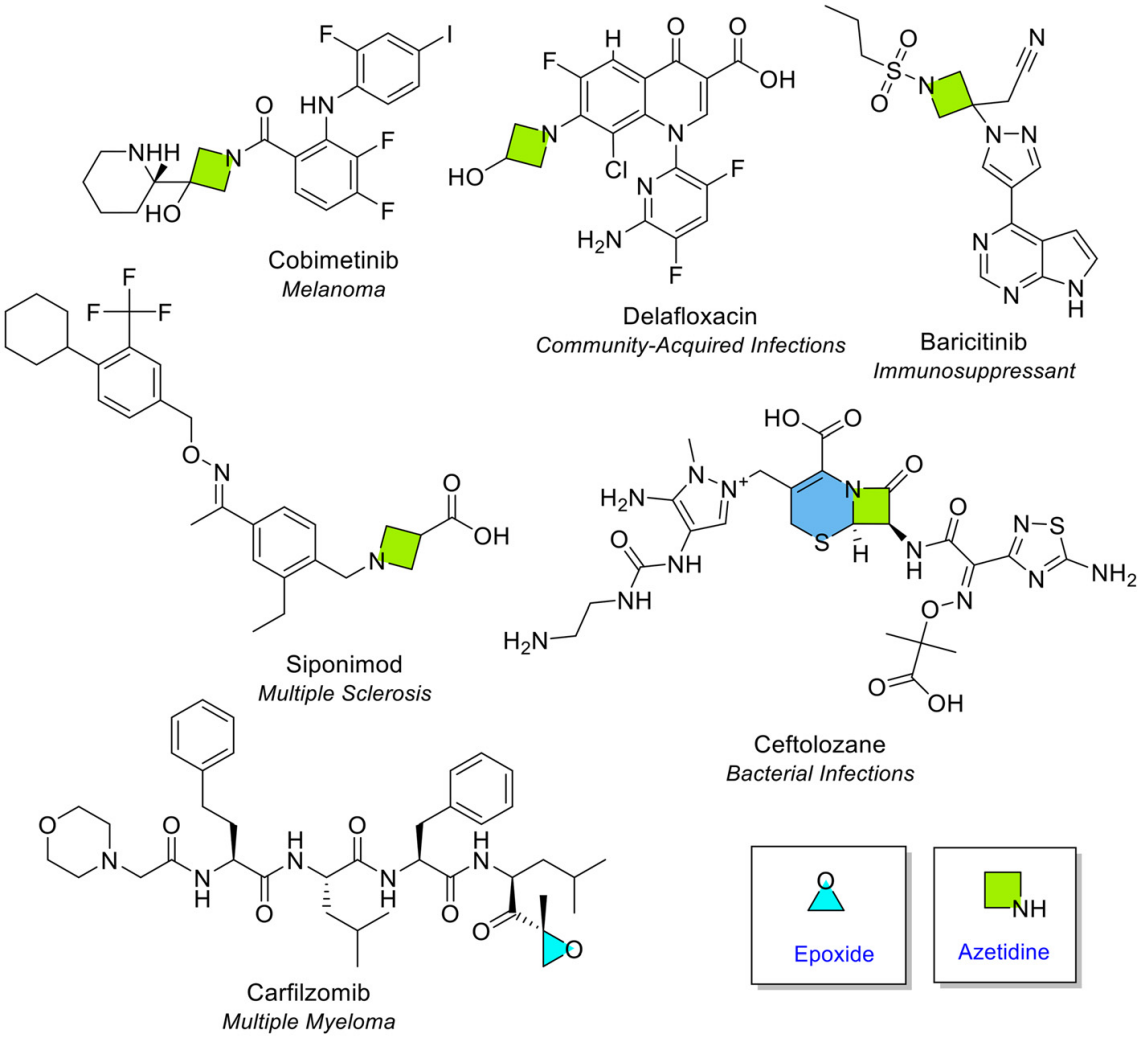
NAS approved by the EMA between 2014–2023 containing epoxide (light blue), azetidine (green) and fused azetidine (blue and green, cepham) heterocycles.

The review of NAS (28 monocyclic heterocycles) in the 10-year period revealed only one 3-membered heterocycle (epoxide) and one 4-membered heterocycle (azetidine) (Fig. 3). Epoxide is a 3-membered ring containing a single oxygen atom and was observed in carfilzomib, a cancer medication used to treat multiple myeloma. Azetidine is a 4-membered saturated ring that contains a single nitrogen atom. Azetidine was seen five times in five different NAS. Two of the azetidine-containing NAS were drugs used for infectious diseases (delafloxacin and ceftolozane). Of the five occurrences of azetidine, four were monocyclic. A cephem-type fused β-lactam ring was noted in ceftolozane. The substitution of the ring varied across each active substance. The azetidine ring was substituted on 2, 3 or 4 positions. The historical influence of beta-lactam containing active substances to treat infectious diseases clearly continues to the present day.

### Five-membered heterocycles amongst EMA approved drugs

**Fig. 4:**
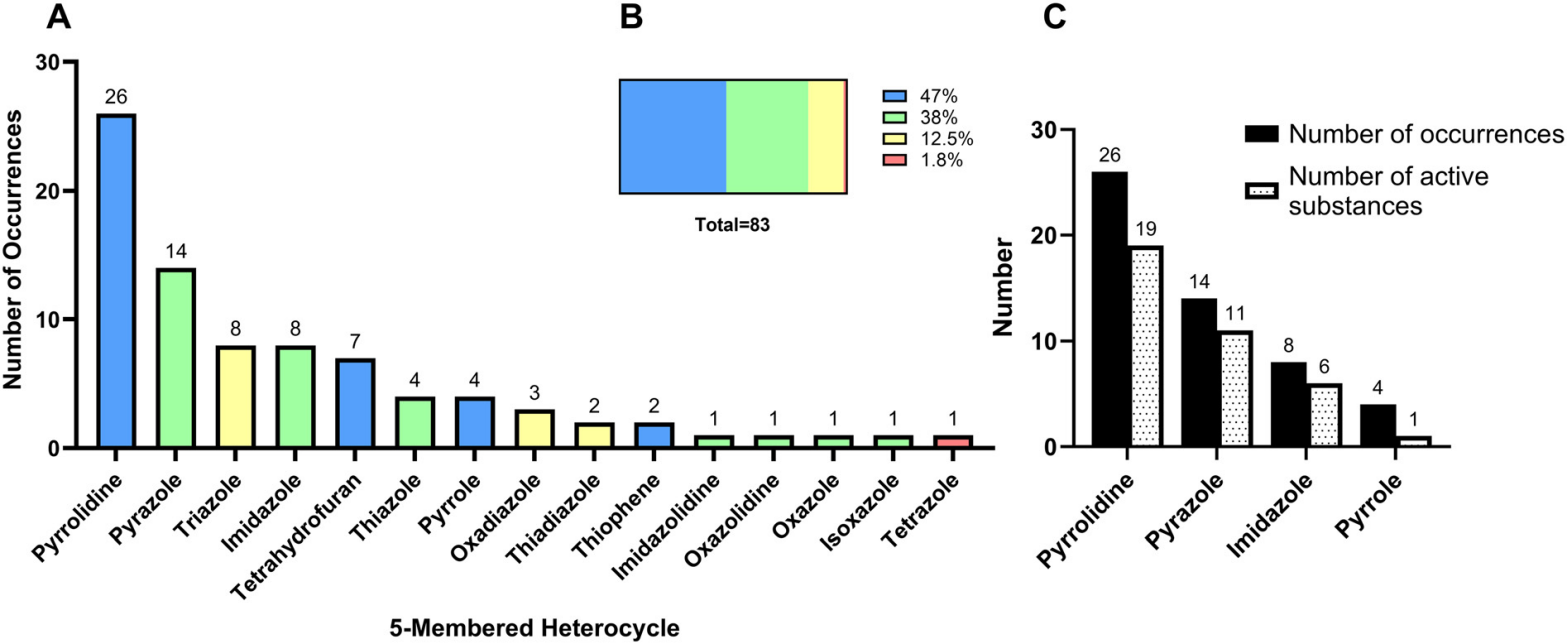
Analysis of 5-membered heterocycles in EMA approved drugs (2014–2023). A. Number of occurrences of monocyclic 5-membered heterocycles in NAS. B. Analysis of all occurrences of 5-membered heterocycle by number of heteroatoms per heterocycle. Colour key for A and B: blue: contains one heteroatom; green: contains two heteroatoms; yellow: contains three heteroatoms; red: contains four heteroatoms. C. 5-Membered heterocycles with multiple occurrences in one NAS.

The review of the 164 NAS in the 10-year period revealed 15 unique 5-membered heterocycles (Fig. 4A). This accounts for 54% of the 28 heterocycle types identified during the initial screening. There is substantial variety within the 5-membered rings due to ring saturation (saturated and unsaturated), multiple heteroatoms in the ring (Fig. 4B), isomers, and multiple ring substitution patterns. Pyrrolidine was by far the most popular 5-membered heterocycle appearing in drug scaffolds (26 occurrences), followed by pyrazole (14 occurrences) (Fig. 4A).

#### 5-Membered heterocycles containing one heteroatom

5-Membered heterocycles that contain a single heteroatom were the most common, occurring 39 times across all NAS (Fig. 4B, blue). Of the 15 five-membered heterocycles, four ring types contained one heteroatom. Of these, two heterocycles contained nitrogen (pyrrolidine and pyrrole, saturated and unsaturated), one contained oxygen (tetrahydrofuran, saturated) and one contained sulfur (thiophene, unsaturated).

##### Pyrrolidine and pyrrole

**Fig. 5:**
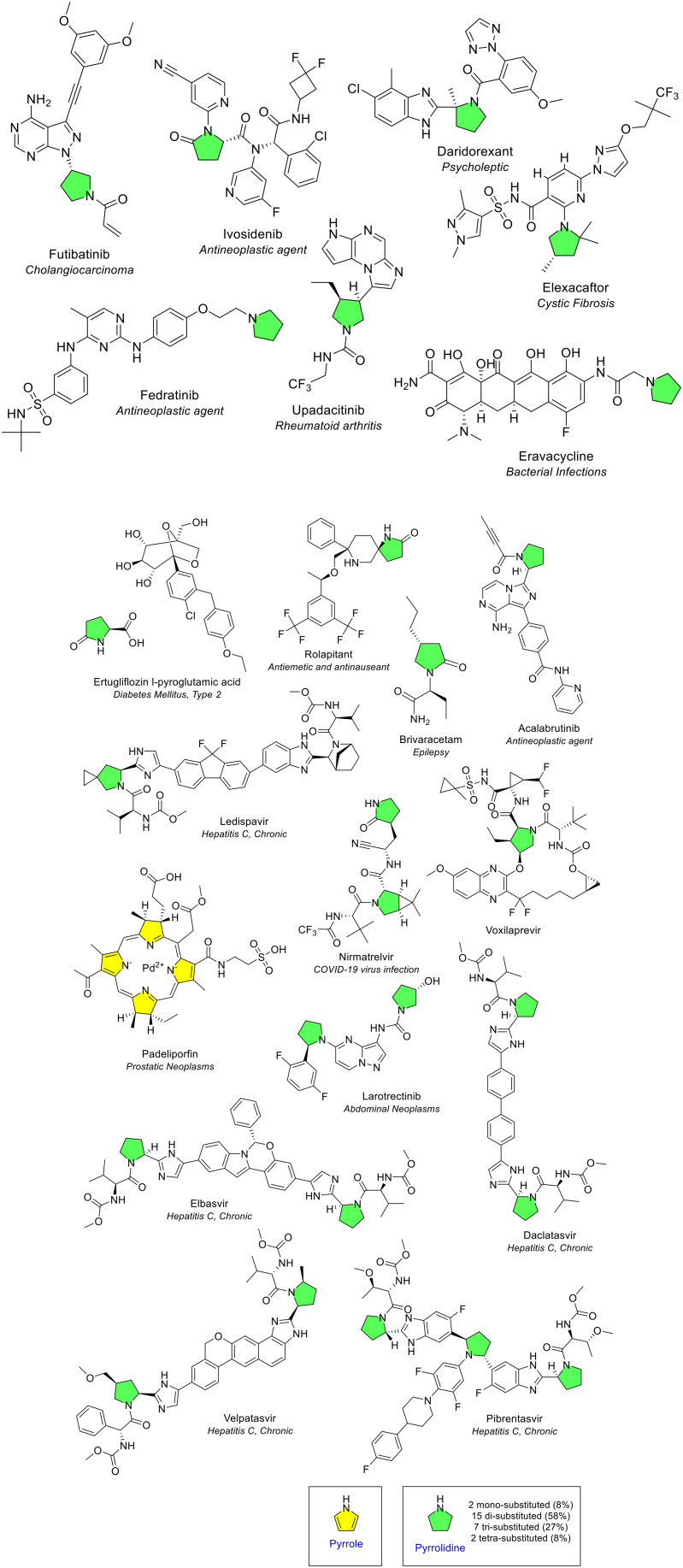
NAS approved by the EMA between 2014–2023 containing pyrrolidine (green) and pyrrole (yellow) heterocycles.

Pyrrolidine was the most common 5-membered heterocycle with one heteroatom, present 26 times across 19 NAS (including in the oxidised pyrrolidine-2-one form) (Fig. 4C). A single pyrrolidine ring was present in 13 NAS, five NAS contained two pyrrolidine rings, and one NAS contained three pyrrolidine rings (green, Fig. 5). One of the NAS, the diabetes medicine ertugliflozin l-pyroglutamic acid, contains the pyrrolidine moiety in the acidic co-crystal. Pyrrolidine is commonly referred to as “*one of the most important heterocyclic compounds*”.^11^ Its presence in medicines can be seen in the majority of therapeutic areas such as drugs for cancer and various infectious diseases. Pyrrolidine was the most common 5-membered heterocycle amongst FDA approved drugs.^4,5^ An oxidised pyrrolidine ring (pyrrolidone) can be seen in a spiro arrangement with a piperidine ring in rolapitant, technically known as a 1,7-diazaspiro[4.5]decan-2-one ring. Ledispavir also contains a pyrrolidine ring in a spiro arrangement with a cyclopropane ring, known as 5-azaspiro[2.4]heptane.

The substitution of the pyrrolidine ring was noted at 1, 2, 3 or 4 positions (Fig. 5). Pyrrolidine rings occur frequently as they are good nucleophiles.^12^ The ring can be utilised as a scaffold and through manipulation of the ring, the physicochemical, toxicological and potency of drugs can be modified and enhanced. Within biological target binding sites, the NH could act as a hydrogen bond donor; if the ring nitrogen is substituted, it has potential to act as a hydrogen bond acceptor. It is notable that the pyrrolidine ring nitrogen was commonly substituted (in 88% of heterocycles, Fig. 5), similar to FDA approvals (92% and 94% respectively for the 2014 and 2024 perspectives). Larotrectinib is a pyrrolidine-containing NAS that was the first ‘histology-independent’ treatment in the EU for solid tumours with a neurotrophic tyrosine receptor kinase (NTRK) gene fusion. It also contains a pyrazolo[1,5-*a*]pyrimidine fused ring system. Pyrrole is structurally similar to pyrrolidine but is unsaturated. Pyrrole was only seen as part of a larger tetrapyrrole ring structure in padeliporfin, which is the NAS in the anti-cancer drug Tookad (yellow, Fig. 5). Pyrrole was generally fused to one or more rings (discussed in the following sections).

##### Tetrahydrofuran

**Fig. 6:**
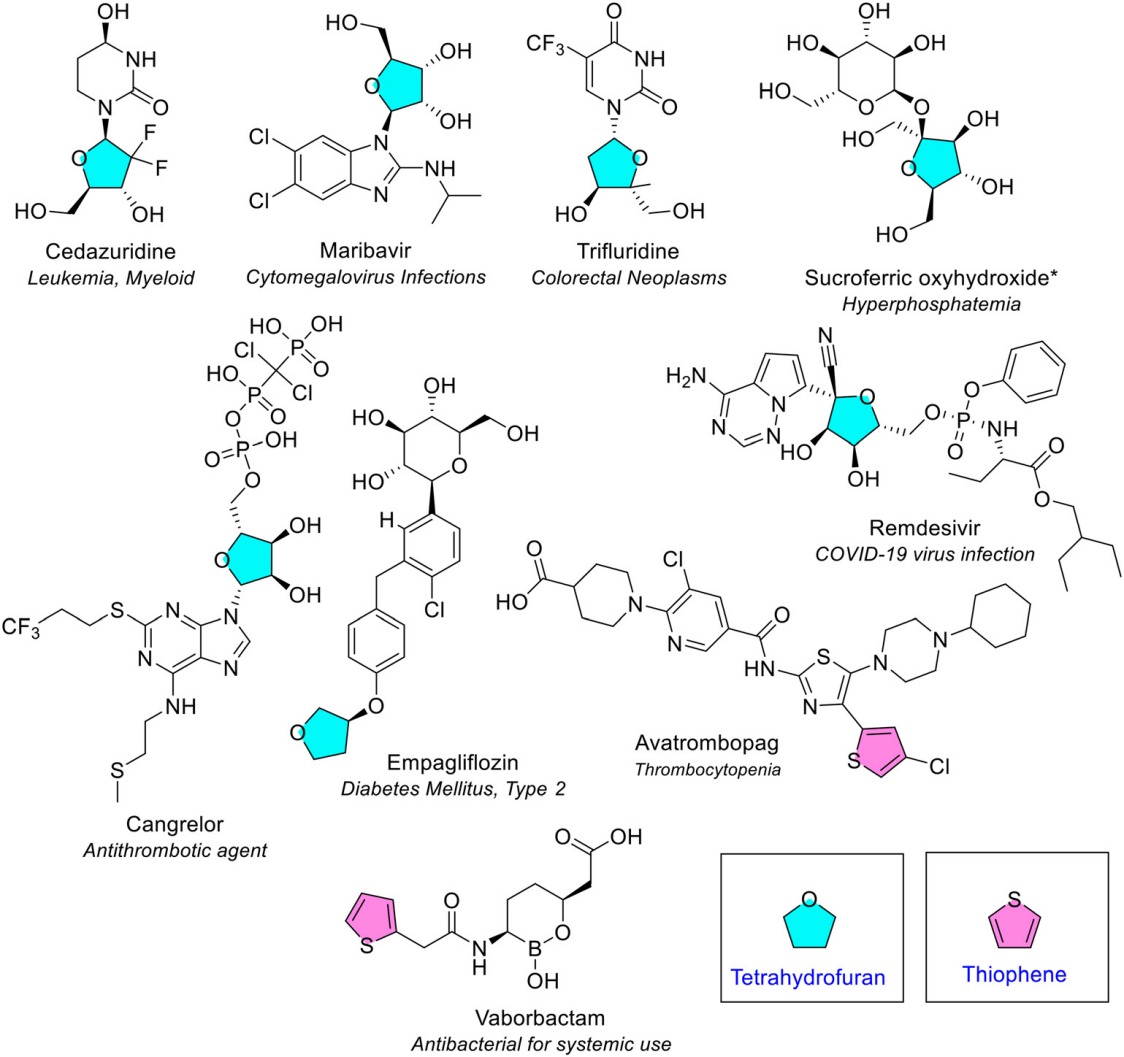
NAS approved by the EMA between 2014–2023 containing tetrahydrofuran (blue) and thiophene heterocycles (pink). *Sucroferric oxyhydroxide is a mixture of polynuclear iron(iii)-oxyhydroxide, sucrose (sugar) and starches; only sucrose is depicted here.

Tetrahydrofuran was the second most common 5-membered heterocycle with one heteroatom. It consists of a single oxygen in a saturated ring and is also known as ‘oxolane’ in IUPAC nomenclature. It was present seven times in seven different NAS each containing one tetrahydrofuran ring (Fig. 4A and blue, Fig. 6). The ring was substituted at 2, 3 or 4 positions. Many of these drugs contain tetrahydrofuran rings that are structurally similar to pentose sugars. For example, sucroferric oxyhydroxide is used to control blood-phosphate levels in patients with long-term kidney disease. Maribavir is a tetrahydrofuran-containing first-in-class human CMV pUL97 viral protein kinase inhibitor, used for treatment of cytomegalovirus infections (also containing a benzimidazole).

##### Thiophene

Thiophene is a sulfur-containing unsaturated ring and was the least common 5-membered heterocycle with one heteroatom of those appearing in EMA approved drugs in the review period. A single thiophene ring was present in two different NAS (Fig. 6). One thiophene ring was monosubstituted (in the antibacterial NAS vaborbactam), and disubstitution was seen in the other NAS (in avatrombopag, used for thrombocytopenia).

#### 5-Membered heterocycles containing two heteroatoms

The second most common type of 5-membered heterocycles were those that contained two heteroatoms, observed 32 times across all NAS. Most of the variety amongst 5-membered rings was seen in this subclass. Of the 15 unique 5-membered heterocycles, 7 ring types contained two heteroatoms (Fig. 4A and B, green). Three of these heterocycles contained two nitrogen atoms (1 saturated and 2 unsaturated rings), three contained one nitrogen and one oxygen (1 saturated and 2 unsaturated rings), and one heterocycle contained one nitrogen and one sulfur atom.

##### Imidazole, pyrazole and imidazolidine

**Fig. 7:**
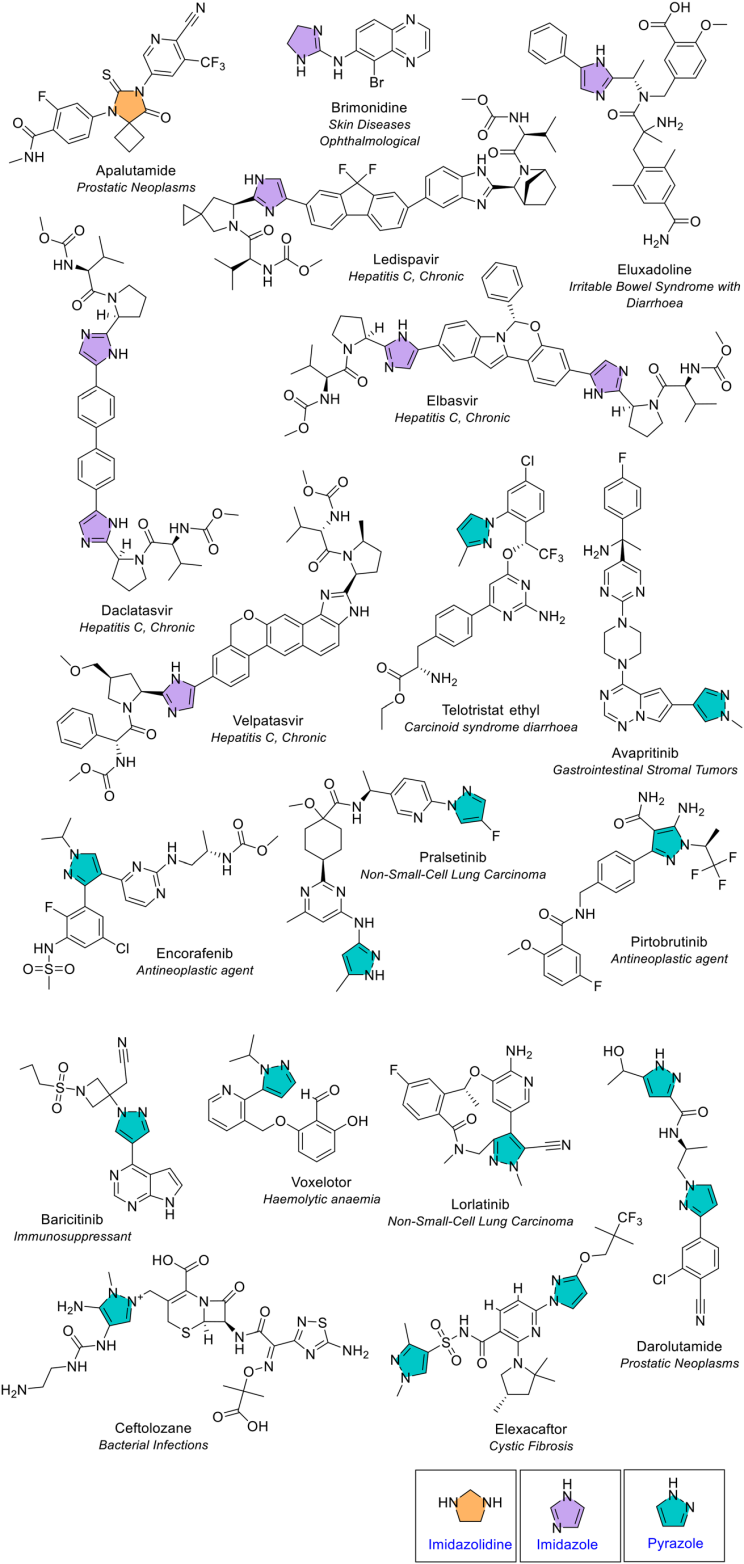
NAS containing imidazolidine (orange), imidazole (purple) and pyrazole (turquoise) monocyclic heterocycles approved by the EMA between 2014–2023.

Imidazole and pyrazole are isomers. Imidazole contains the two nitrogen atoms in the 1,3 position, whilst pyrazole contains two nitrogen atoms in the 1,2 position. Pyrazole was the most common 5-membered heterocycle with two heteroatoms and was present 14 times across 11 different NAS (Fig. 4A, C and 7). A single pyrazole ring was present in eight NAS, and three NAS contained two pyrazole rings. The variety of substitution seen on the pyrazole ring was diverse, with substitution at 2, 3 or 4 positions. Imidazole was present eight times across six different NAS. A single imidazole ring was present in four NAS, and two NAS contained two imidazole rings. The substitution of the imidazole ring varied as either monosubstituted or disubstituted (Fig. 7).

Imidazole, alongside benzimidazole, is an electron-rich heterocycle that can accept or donate protons, and also form diverse weak interactions easily, contributing to its ability to bind to a diverse range of biological targets.^13^ Likewise, pyrazole is considered as a privileged scaffold in drug discovery.^14^ The original review of heterocycles in FDA-approved drugs noted imidazole as the third most common 5-membered heterocycle.^4^ The latest review noted a higher count of imidazole (20 appearances in 18 active substances) and pyrazole (20 appearances).^5^ There are several potential reasons for the differences in FDA *versus* EMA data. Firstly, the FDA review period covered 11 years (January 2013 – December 2023) compared to 10 years in our study and naturally several different medicines were approved by both agencies. Secondly, there were differences in classification of fused heterocycles. FDA data included several uncommon fused rings as imidazoles (*e.g.* those in osilodrostat, remimazolam, pretomanid and quizartinib), whereas we classified all fused analogues separately (*e.g.* osilodrostat was classified as a bicyclic heterocycle containing a pyrrolo[1,2-*c*]imidazole core). The peptides afamelanotide and setmalonotide were also not included in our data.

Imidazolidine is a 5-membered saturated ring consisting of 2 nitrogen atoms in the 1,3 position. Imidazoline was seen only once (Fig. 4). It is described as a labile ring that is susceptible to hydrolysis.^15^ To overcome this, imidazolidine rings often are fused to one or more rings. The only NAS that contained an imidazolidine ring was apalutamide which is the NAS in the anti-cancer drug Erleada (Fig. 7). Even in this case, the imidazolidine ring was oxidised and in a spiro arrangement technically known as a 5,7-diazaspiro[3.4]octane ring.

##### Oxazolidine, oxazole and isoxazole

**Fig. 8:**
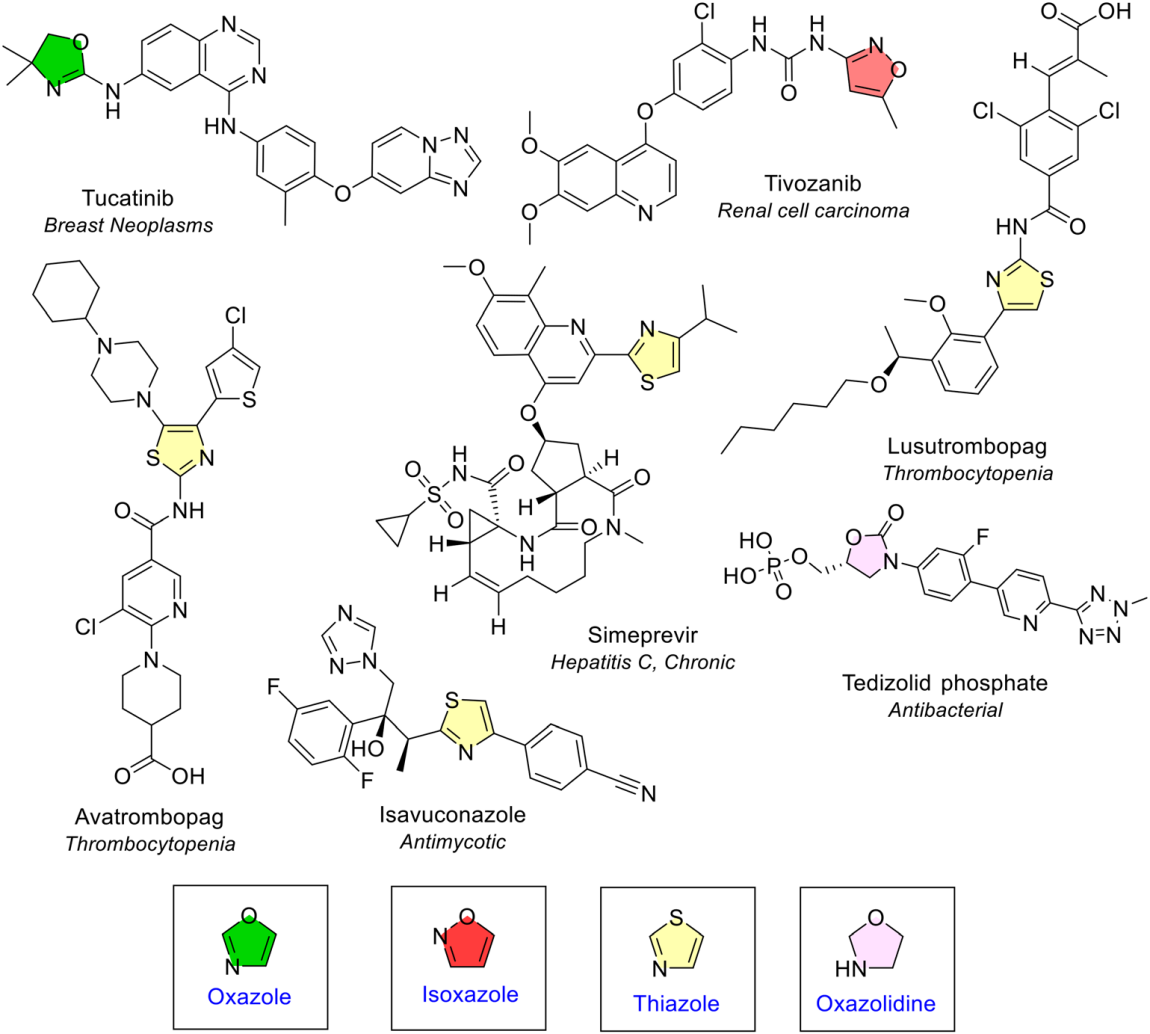
NAS approved by the EMA between 2014–2023 containing oxazolidine (pink), oxazole (green), isoxazole (red), and thiazole (yellow) heterocycles.

Oxazolidine is a saturated 5-membered ring containing nitrogen and oxygen in the 1,3 position. Oxazolidine was observed once in the antibiotic tedizolid phosphate (Fig. 4A and 8). Oxazole and isoxazole are 5-membered rings that contain a nitrogen and oxygen atom. Oxazole is the 1,3 isomer and isoxazole the 1,2 isomer. Both were only observed once; oxazole in tucatinib (for treatment of breast cancer) and isoxazole in tivozanib (for treatment of advanced renal cell carcinoma) (Fig. 4A and 8).

##### Thiazole

Thiazole is an unsaturated 5-membered rings that contains nitrogen and sulfur in the 1,3 position. Thiazole was the third most common 5-membered heterocycle with two heteroatoms (Fig. 4A). It was present four times across four different NAS and was either disubstituted or trisubstituted (Fig. 8).

#### 5-Membered heterocycles containing three or four heteroatoms

5-Membered heterocycles containing three heteroatoms were observed 13 times across all NAS (Fig. 4A and B, yellow). Of the 15 five-membered heterocycles, three different ring types contained three heteroatoms. Of these three ring types, one of these contained three nitrogen atoms (triazole; unsaturated), one contained two nitrogen atoms and one oxygen atom (oxadiazole; unsaturated), and one contained two nitrogen and one sulfur atoms (thiadiazole; unsaturated).

##### Triazole

**Fig. 9:**
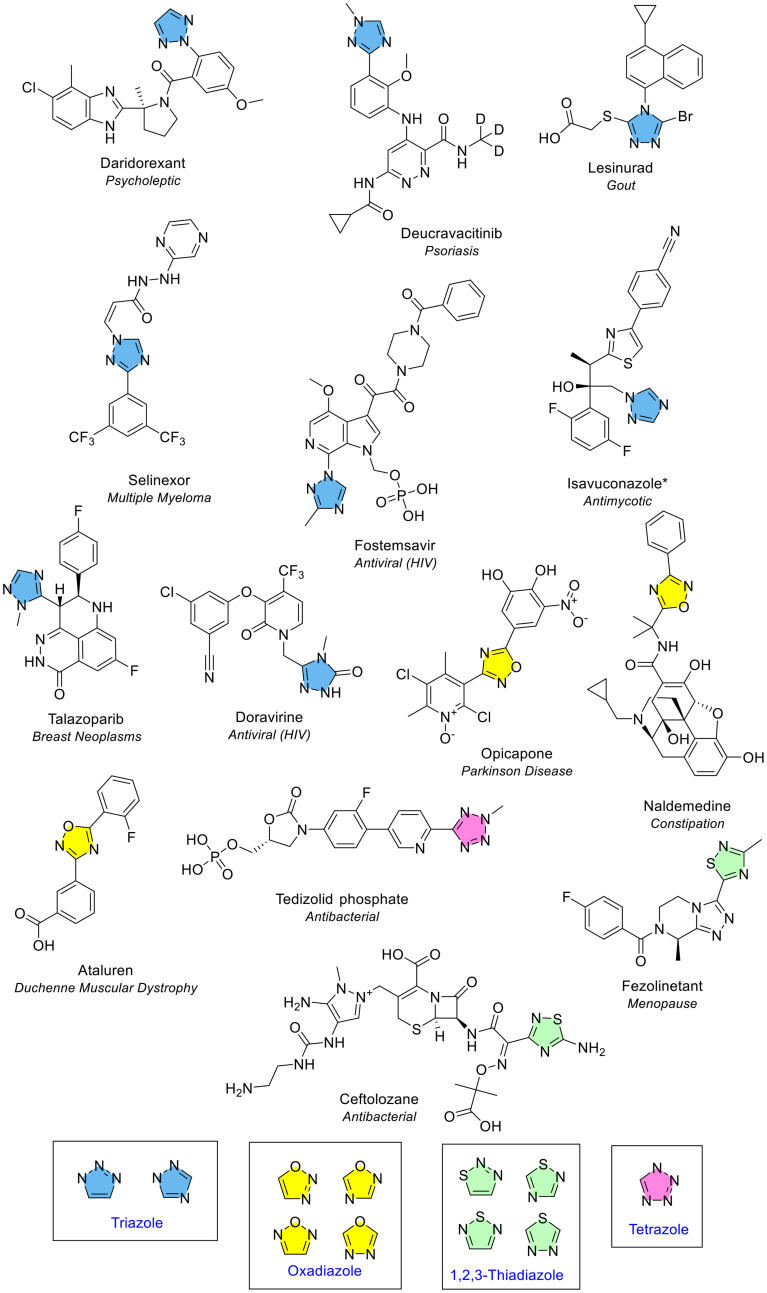
NAS containing monocyclic 5-membered heterocycles with three or four heteroatoms approved by the EMA between 2014–2023; key: triazole (blue), oxadiazole (yellow), thiadiazole (green) and tetrazole (pink). *Isavuconazole is approved in the form isavuconazonium sulfate.

Triazole is an unsaturated 5-membered ring which contains three nitrogens and was the most common 5-membered heterocycle containing three heteroatoms. Triazoles have distinct physiochemical properties including, for example, lower log *P* than phenyl rings and lower p*K*_a_ compared to pyridine and imidazole.^16^ It was present in eight different NAS (Fig. 4 and 9). The recent review of FDA approved drugs noted nine occurrences of 1,2,4 triazole.^5^ Triazole exists as two isomers with the difference due to the location of the nitrogen atoms on the 5-membered ring (1,2,3 or 1,2,4). Both isomers can exist as two tautomers. Of the eight EMA approvals, one (daridorexant) was the 1,2,3 isomer and seven were the 1,2,4 isomer (blue, Fig. 9). Daridorexant is a dual oxrexin receptor antagonist (DORA), used to treat insomnia. Suvorexant was the first-in-class DORA, also containing the 1,2,3 triazole isomer, and although it is licensed in several countries including the US, it is not yet available in the EU. Deucravacitinib is a deuterated 1,2,4-triazole-containing NAS approved in 2023 for treating moderate to severe plaque psoriasis.

##### Oxadiazole and thiadiazole

Oxadiazole is an unsaturated 5-membered ring consisting of two nitrogen and one oxygen atoms. It was present three times in three different NAS (Fig. 4A). Oxadiazole has four possible isomers, but all occurrences were the 1,2,4 isomer (yellow, Fig. 9). Thiadiazole is an unsaturated 5-membered ring which contains two nitrogen and one sulfur atoms. It occurred twice and, as for oxadiazole, both NAS were the 1,2,4 structural isomer (green, Fig. 9). All occurrences of oxadiazole and thiadiazole were disubstituted.

##### Tetrazole

Tetrazole was the only 5-membered heterocycle that contained four heteroatoms (all nitrogens) (Fig. 4A, red). It was present in the antibiotic tedizolid phosphate (pink, Fig. 9). Tetrazole has three positions that can be substituted; however, it can only have a maximum of two substitutions at a time.^4^

### 6-Membered heterocycles amongst EMA approved drugs

**Fig. 10:**
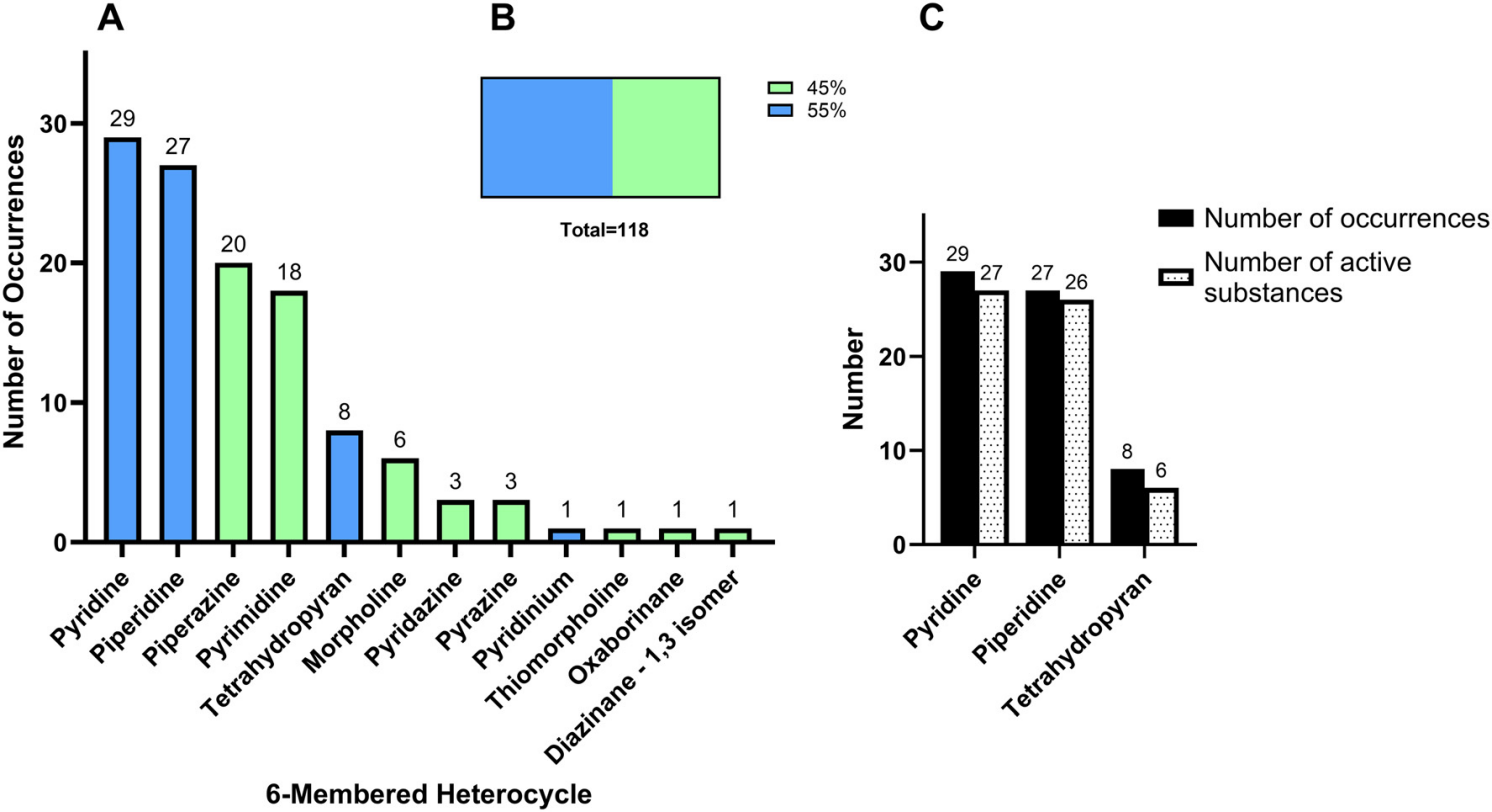
Analysis of 6-membered heterocycles in EMA approved drugs (2014–2023). A. Number of occurrences of monocyclic 6-membered heterocycles in NAS. B. Analysis of all occurrences of 6-membered heterocycle by number of heteroatoms per heterocycle. Colour key for A and B: blue: contains one heteroatom; green: contains two heteroatoms. C. 6-Membered heterocycles with multiple occurrences in one NAS.

The review of the 164 NAS in the 10-year period revealed 11 unique 6-membered heterocycles (Fig. 10A). Of the 28 monocyclic heterocycle types identified, 39% were 6-membered rings. The diversity amongst the 6-membered rings is similar to 5-membered rings, arising from ring saturation (saturated and unsaturated), multiple heteroatoms on the ring, isomers, and different substitution patterns. Of 119 total occurrences of 6-membered heterocycles, 55% contain one heteroatom, 45% contain two heteroatoms, and no rings contain three or more heteroatoms (Fig. 10B). There were fewer types of six-membered heterocycles containing one heteroatom, but they were more numerous due to the popularity of pyridine and piperidine in drug scaffolds.

#### 6-Membered heterocycles containing one heteroatom

6-Membered heterocycles that contain a single heteroatom were very common, seen a total of 65 times. Of the 11 unique 6-membered heterocycles, four contained one heteroatom (pyridine, piperidine, tetrahydropyran and pyridinium, Fig. 10A). Three of these heterocycles contained one nitrogen atom (1 saturated and 2 unsaturated rings) and one heterocycle contained one oxygen atom (saturated; tetrahydropyran).

##### Pyridine and pyridinium

**Fig. 11:**
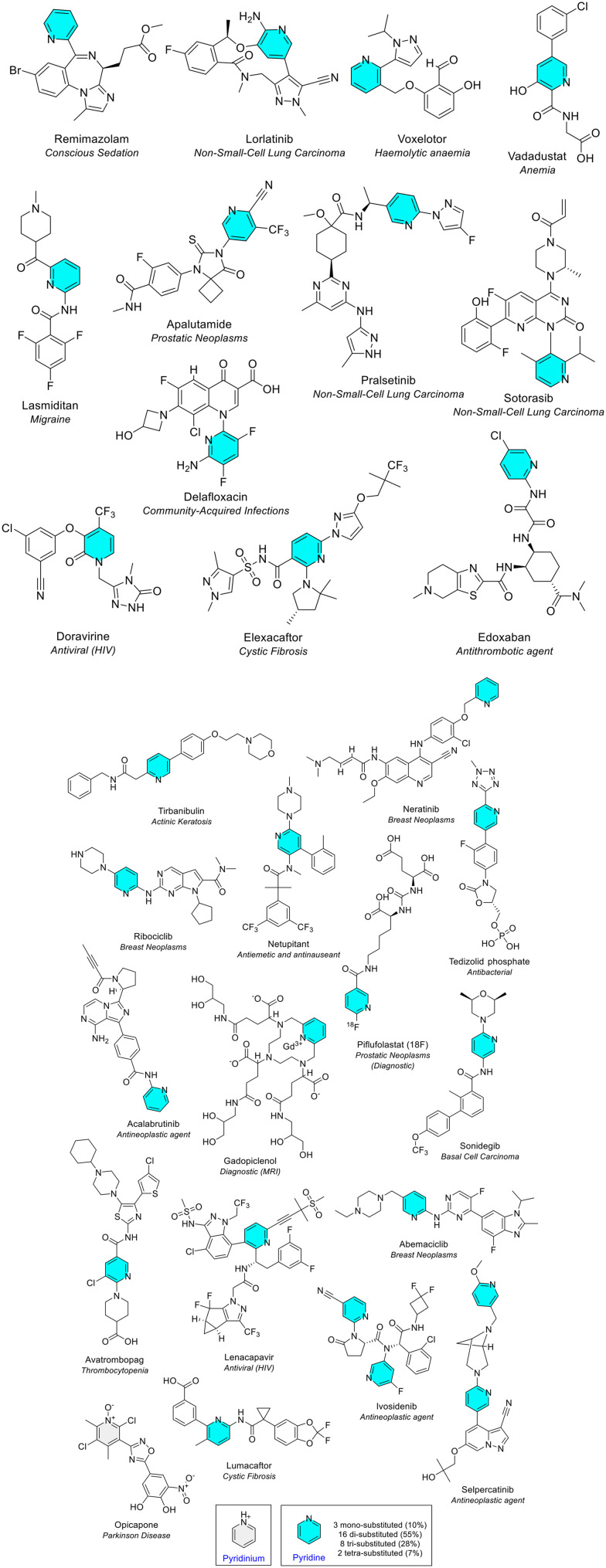
NAS approved by the EMA between 2014–2023 containing pyridine (blue) and pyridinium (grey) heterocycles.

Pyridine and pyridinium both contain a single nitrogen atom in a 6-membered unsaturated ring. The nitrogen atom possesses a non-bonding electron pair which can form hydrogen bonds with biological targets. Pyridine is incorporated in drugs for diverse clinical indications.^17^ Pyridine was the most common 6-membered heterocycle containing one heteroatom. It was seen 29 times across 27 different NAS (Fig. 10A, C and 11). A single pyridine ring was seen in 25 NAS, and two NAS (selpercatinib and ivosidenib) contained two pyridine rings (Fig. 11). Gadopiclenol is a contrast agent for MRI that contains pyridine as part of a larger pyclen-based structure, as well as the rare earth metal gadolinium. The radiopharmaceutical piflufolastat (^18^F), approved in 2023, is also a diagnostic tool used detect prostate cancer cells using positron-emission tomography (PET). The antiviral NAS doravirine contains an oxidised pyridine ring. Pyridine ring substitution occurred at 1, 2, 3 or 4 positions. Di-substitution was the most common (16 occurrences, 55%) (Fig. 11). Pyridine was also popular in FDA approved drugs, as the second most common 6-membered ring in the earlier review and the most common in the more recent review (54 occurrences).^4,5^ The difference in the number of pyridine occurrences in EMA data compared to the recent FDA data may be explained by several factors: different drug approvals, combination of pyridine and pyridinium as one category in the FDA review, and counting of fused rings containing pyridine in the FDA review [*e.g.* atogepant, ubrogepant, lonafarib, and flortaucipir (^18^F)]. There is a huge variety of pyridine-fused ring types as discussed later. Pyridinium is the conjugate acid of pyridine. A single hexa-substituted pyridinium ring was observed in the NAS opicapone, which is used as an add-on to levodopa or DOPA decarboxylase inhibitors to treat adults with Parkinson's disease.

##### Piperidine

**Fig. 12:**
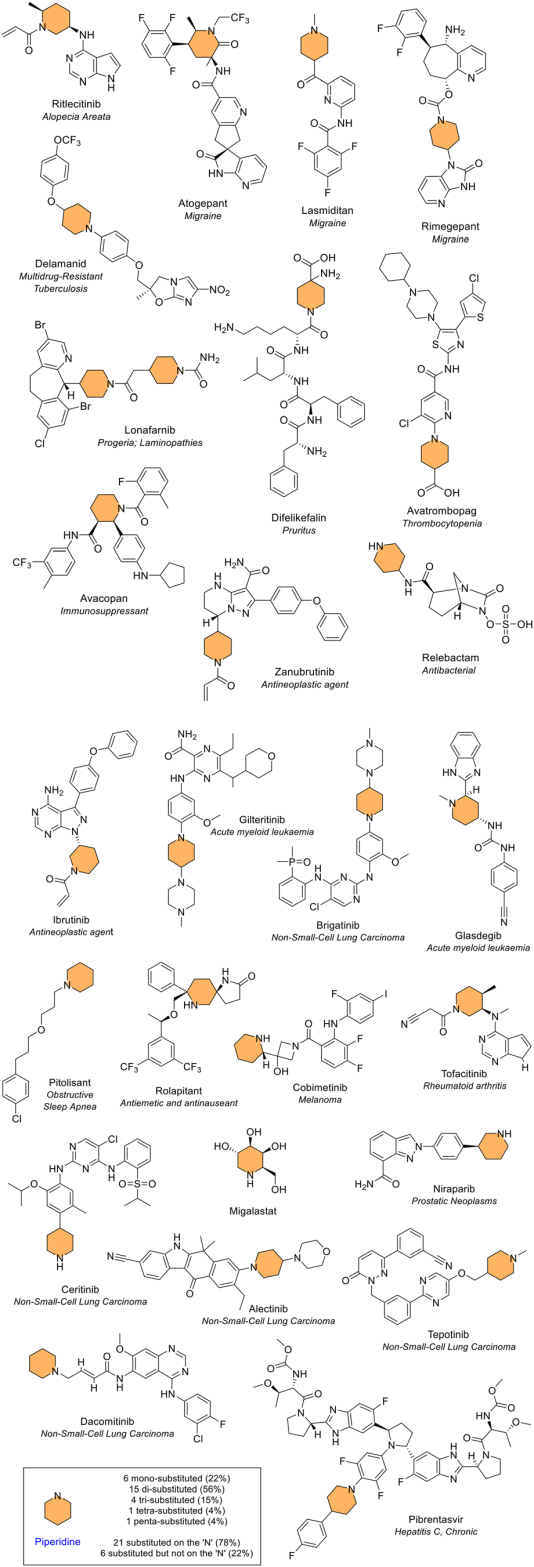
NAS approved by the EMA between 2014–2023 containing a piperidine (orange) heterocycle(s).

Piperidine is a saturated 6-membered ring that contains a single nitrogen atom and is a common fragment in drug design.^18^ Like pyrrolidine, it is an important heterocyclic compound. Incorporation of a piperidine ring can impact pharmacokinetic properties such as lipophilicity and metabolic stability. In this analysis, piperidine was the second most common 6-membered heterocycle containing one heteroatom, present 27 times across 26 different NAS (Fig. 10A and C). In 78% of occurrences, the piperidine moiety was substituted on the ring nitrogen. One NAS, the first-in-class farnesyl transferase (FTase) inhibitor lonafarnib, contained two piperidine rings (Fig. 12). The substitution of the piperidine ring varied across the NAS, with substitution at 1, 2, 3, 4 or 5 positions (Fig. 12). Rolapitant contained the piperidine ring in a spiro configuration (1,7-diazaspiro[4.5]decan-2-one ring), approved as an antiemetic for cancer chemotherapy that has since been withdrawn at the request of the marketing authorisation holder. Avacopan (Fig. 12) was a first-in-class medicine approved in 2021 to treat adults with either of two forms of a rare multisystem autoimmune condition: severe, active granulomatosis with polyangiitis or microscopic polyangiitis. The initial review of FDA approved drugs found piperidine as the most common 6-membered ring, whilst the more recent review noted it as the second most common 6-membered heterocycle.^4,5^*N*-Benzyl piperidine is a common fragment observed in five of the NAS (Fig. 12), typically included in drug scaffolds due to its structural flexibility and ability to provide cation-π interactions with biological targets.^19^

##### Tetrahydropyran

**Fig. 13:**
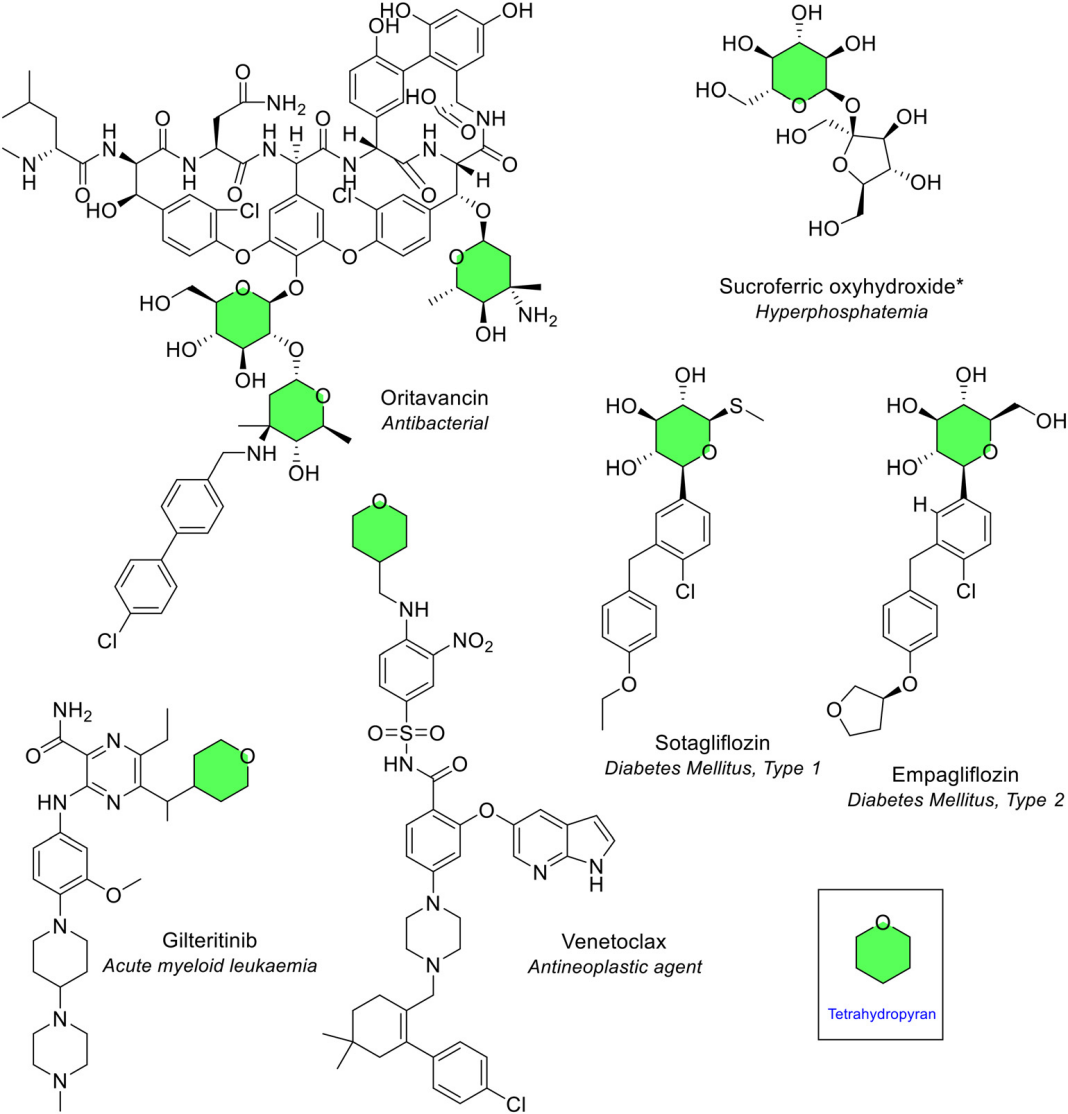
NAS approved by the EMA between 2014–2023 containing a tetrahydropyran (green) heterocycle. *Sucroferric oxyhydroxide is a mixture of polynuclear iron(iii)-oxyhydroxide, sucrose (sugar) and starches; only sucrose is depicted here.

Tetrahydropyran is a saturated 6-membered ring that contains a single oxygen atom. The tetrahydropyran ring is known as “oxane” by IUPAC naming conventions. This analysis revealed tetrahydropyran as the third most common 6-membered heterocycle containing one heteroatom, seen eight times in six different NAS (Fig. 10A and C). One NAS, the antibiotic oritavancin, contained three tetrahydropyran rings which was unique (Fig. 13). Tetrahydropyrans are often the basis of sugars such as glucose. Many of the six NAS contain a sugar moiety, an example being the pyranose ring seen in sotagliflozin (Fig. 13). Because of tetrahydropyran's structure, the ring can only be substituted on a maximum of five positions. Substitution was noted at 1, 4 or 5 positions. The most common was pentasubstitution (all available positions substituted), observed in four of the six NAS.

#### 6-Membered heterocycles containing two heteroatoms

The second most common 6-membered heterocycles were those that contained two heteroatoms, with 54 occurrences across all NAS. There was considerable variety amongst 6-membered rings in this class of heterocycles as, of the 11 unique 6-membered heterocycles, seven ring types contained two heteroatoms (diazinanes, pyrimidines, morpholine, pyridazines, pyrazines, thiomorpholine and oxaborinane; Fig. 10). Four heterocycles contained two nitrogen atoms (1 saturated and 3 unsaturated rings), one heterocycle contained one nitrogen and one oxygen (saturated; morpholine), one heterocycle contained one nitrogen and one sulfur (saturated and unsaturated), and one heterocycle contained one boron and one oxygen (saturated; oxaborinane).

##### Diazinane

**Fig. 14:**
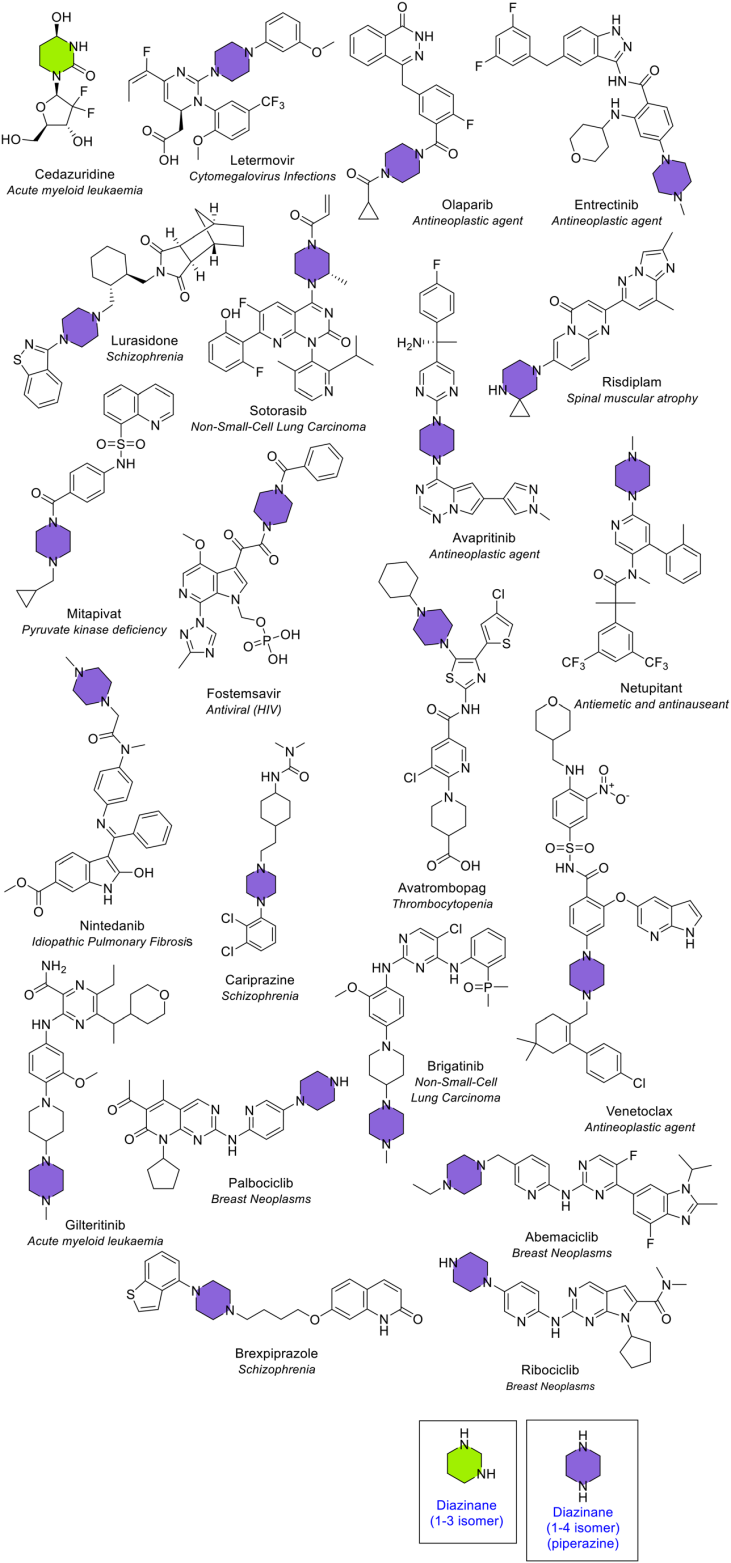
NAS approved by the EMA between 2014–2023 containing a diazinane heterocycle. Cedazuridine contains the 1,3 isomer (green) whilst all other NAS contain the 1,4 isomer (piperazine, purple).

Diazinanes are 6-membered saturated rings containing two nitrogen atoms. They were the most common 6-membered heterocycle that contain two heteroatoms. A diazinane ring was seen 21 times in 21 different NAS (Fig. 10 and 14). The diazinane ring exists in 3 different isomeric forms because of the three possible arrangements of the two nitrogen atoms in the 6-membered ring (1,2, 1,3 or 1,4 position). The 1,2 isomer was not observed. One ring in cedazuridine was the 1,3 isomer (oxidised), and the majority (20) were the 1,4 isomer. The 1,4 isomer of diazinane is more commonly known as piperazine. Piperazine is a common fragment in drugs. The two ring nitrogens offer a large polar surface area and the possibility for hydrogen bond donation and acceptance.^20^ Seventeen (85%) of the piperazine-containing NAS were disubstituted on both ring nitrogens, with the only three exceptions being palbociclib, ribociclib and risdiplam. Risdiplam was the only piperazine-containing NAS with a substituent on a piperazine ring carbon. This substituent was a cyclopropane ring in a spiro arrangement, known in combination as 4,7-diazaspiro[2.5]octane.

Both reviews of FDA-approved drugs also found piperazine as the most common 6-membered heterocycle with two heteroatoms, with no occurrences of the 1,2 or 1,3 isomer noted.^4,5^ Piperazine rings are useful in developing and producing new bioactive molecules.^20^ The inclusion of these rings offers medicinal chemists an easy way to modify the drug's desired pharmacological activity. The two nitrogen atoms in the ring provide a greater polar surface area, as well as more acceptors and donors for hydrogen bonds. These properties are helpful in drug design as they may enhance the drug's pharmacodynamic and pharmacokinetic properties. The diazinane ring occurred several times in different fused configurations, as discussed later.

##### Diazine – pyridazine, pyrimidine and pyrazine

**Fig. 15:**
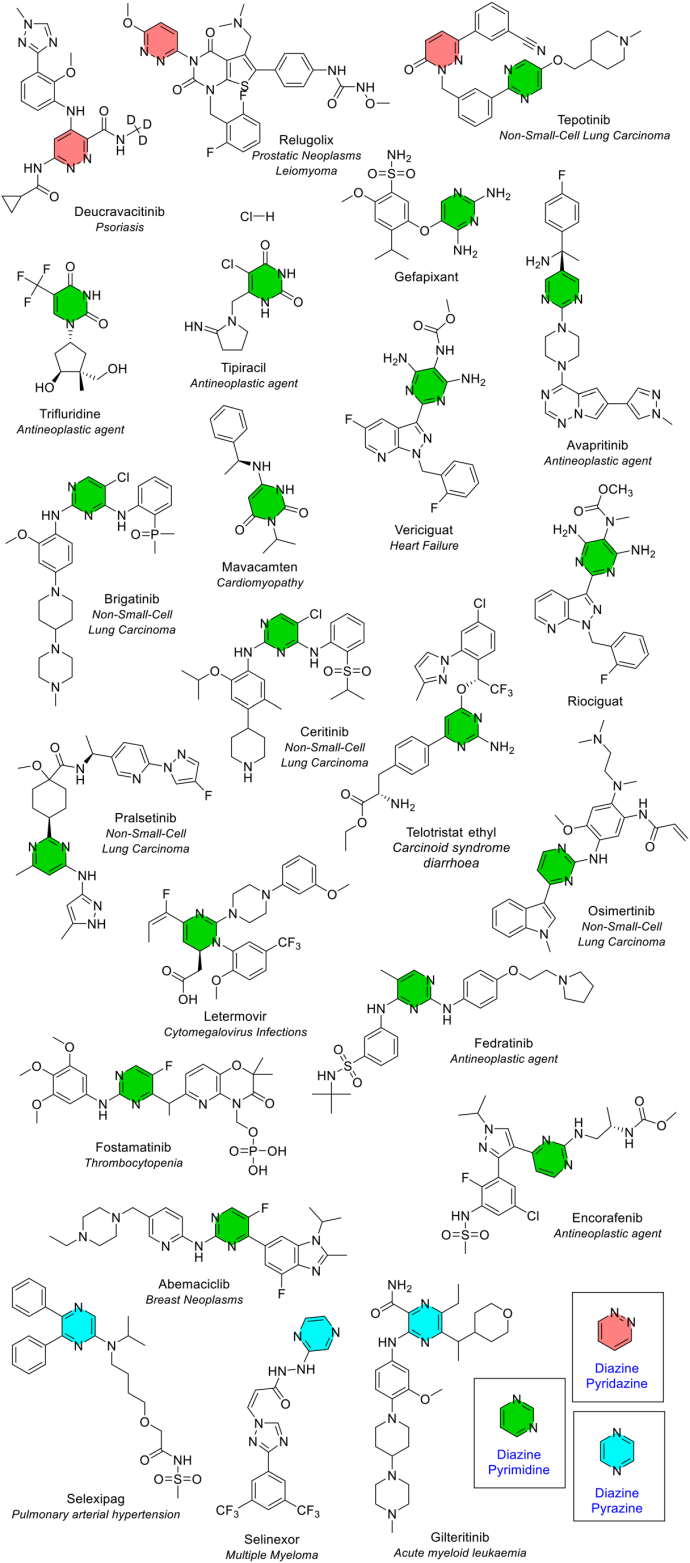
NAS approved by the EMA between 2014–2023 containing a diazine heterocycle; colour key: red = pyridazine (1,2 isomer), green = pyrimidine (1,3 isomer), blue = pyrazine (1,4 isomer).

Diazine is a 6-membered unsaturated ring that contains two nitrogen atoms. Oxidised diazine rings were also included in this section (*e.g.* in the cancer medicine tipiracil). Like the saturated analogue, diazine rings also have 3 structural isomers (1,2, 1,3 or 1,4 isomers). The 1,2 isomer of diazine is known as pyridazine. Compared to the other two isomers, this ring type is rarely found. This heterocycle appeared three times in three NAS (deucravacitinib for plaque psoriasis, and cancer medicines relugolix and tepotinib). All three were also approved by the FDA. Di-substitution was seen twice, and tri-substitution once (Fig. 15). Tepotinib contained both the 1,2 and 1,3 isomer.

The 1,3 isomer of diazine is known as pyrimidine. Pyrimidine was the second most common 6-membered heterocycle that contains two heteroatoms, seen 18 times in 18 different NAS, with substitution at 2, 3, or 4 positions (39%, 44% and 17% for di-substituted, tri-substituted and tetra-substituted respectively, Fig. 15). Several of the NAS contain the RNA base uracil in the core pyrimidine structure (*e.g.* the NAS tipiracil and trifluridine, both components of the medicine Lonsurf for cancer treatment) (Fig. 15). Both FDA reviews found pyrimidine as the second most common 6-membered heterocycle containing two heteroatoms, with trisubstitution the most common in the 2024 perspective.^4,5^ Several medicines included in the FDA data for pyrimidine, for example, adagrasib and palbociclib, are classified within the fused heterocycle section here. The final 1,4 isomer of diazine is known as pyrazine. A pyrazine ring was seen three times in three different NAS with substitution at 1, 2, or 3 positions (Fig. 10 and 15). If all diazine isomers (pyridazine, pyrimidine and pyrazine) were classified together, they would be the most common 6-membered heterocycle containing two heteroatoms (24 times in 23 different NAS).

##### Morpholine

**Fig. 16:**
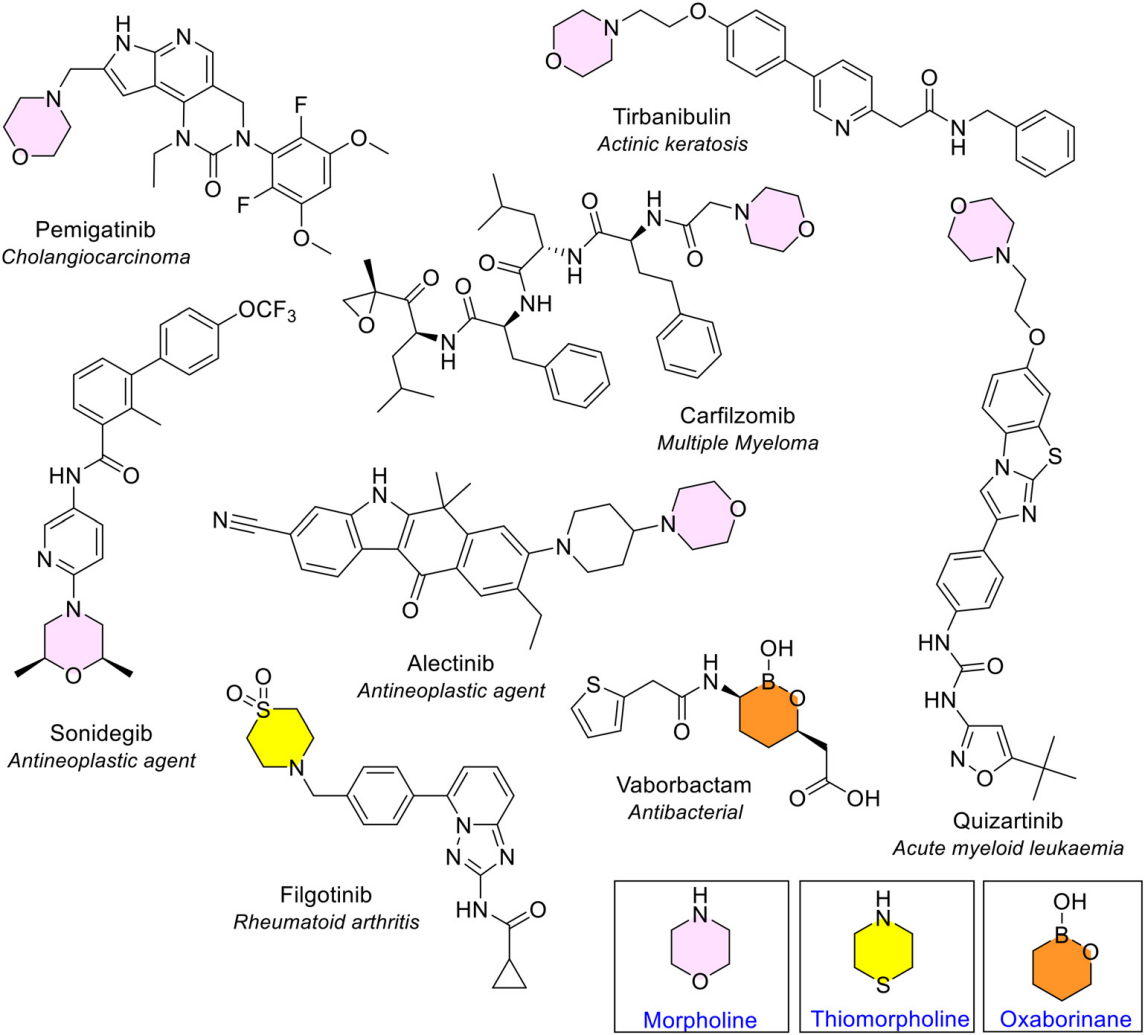
NAS approved by the EMA between 2014–2023 containing morpholine (pink), thiomorpholine (yellow) and oxaborinane (orange) heterocycles.

Morpholine is a saturated 6-membered ring that contains a single nitrogen and oxygen atom in the 1,4 position, which was the third most common 6-membered heterocycle containing two heteroatoms in line with the two previous reviews of FDA data. It was seen six times across six different NAS (Fig. 10 and 16). All six NAS were substituted on the nitrogen atom. The ring was monosubstituted in five of these; trisubstitution was observed once in sonidegib, a medicine used to treat basal cell carcinoma.

##### Thiomorpholine and oxaborinane

Thiomorpholine is a saturated 6-membered ring containing 1 nitrogen and 1 sulfur atom in the 1,4 position, found in only one NAS, filgotinib, in the rheumatoid arthritis drug Jyseleca (Fig. 16). The oxaborinane ring can be considered one of the most unique saturated 6-membered rings, containing one oxygen and one boron atom in the 1,2 position and occurring only once in vaborbactam (Fig. 16). This is one of the NAS in Vaborem, an antibiotic that is used to treat bacteraemia.

### Diversity of fused heterocycles amongst EMA approved drugs

**Fig. 17:**
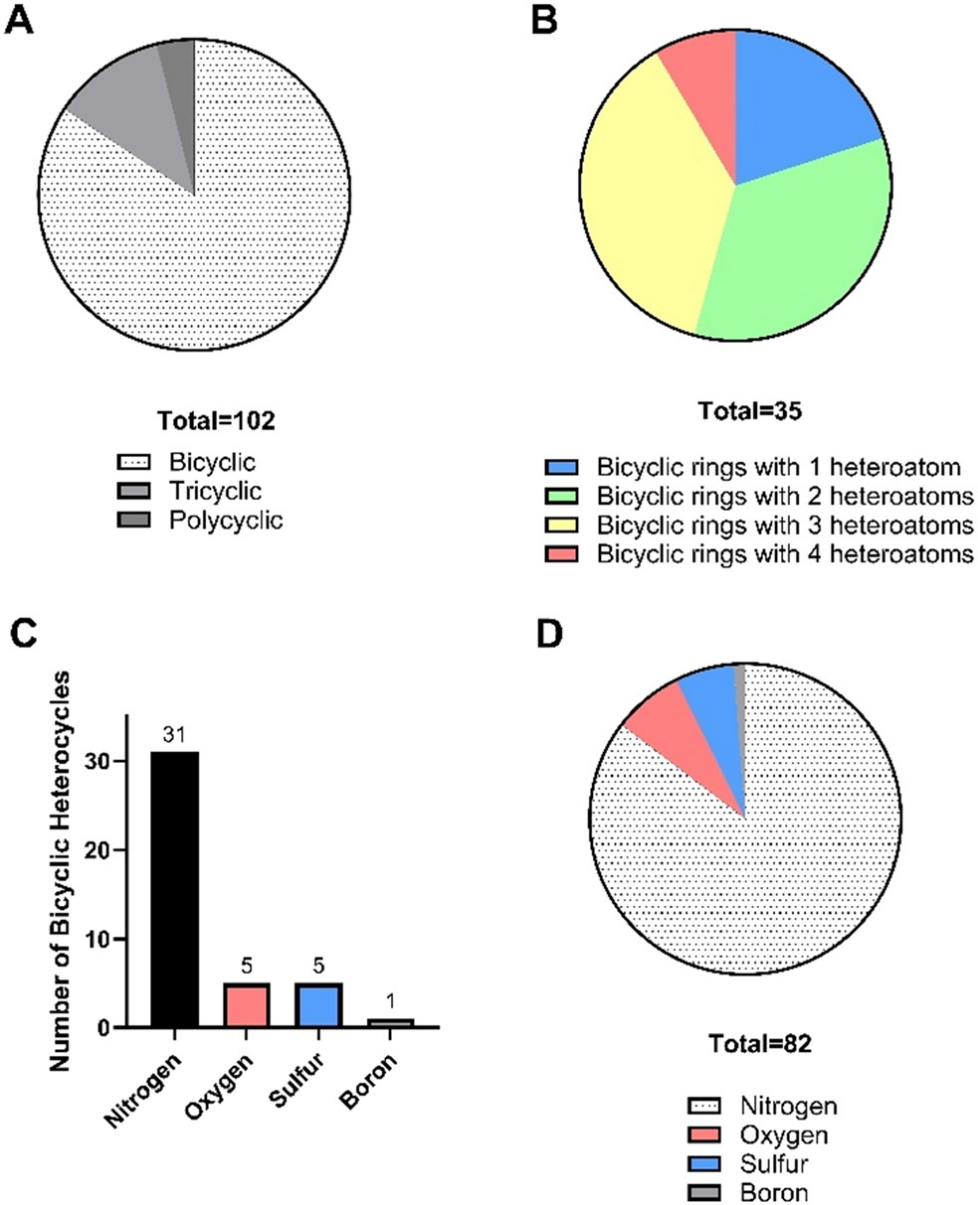
A. Breakdown of NAS approved by the EMA between 2014–2023 by presence of a bicyclic, tricyclic or polycyclic (4 + 5 ring) system; B. analysis of NAS containing bicyclic heterocycles by number of heteroatoms per ring. Note that several NAS contain more than one fused heterocycle; C. analysis of presence of heteroatoms in bicyclic heterocycles (each atom type counted once, *e.g.* pyridopyrimidine contains three nitrogens, counted as one N); D. analysis of all occurrences of atoms in bicyclic heterocycles (*e.g.* pyridopyrimidine contains three nitrogens, counted as three N).

Of the 164 heterocycle-containing NAS approved by the EMA in the 10-year period 2014–2023, the majority (96) contained at least one fused heterocycle (59%), whilst 68 NAS contained only one or more monocyclic heterocycles (41%) (Fig. 1C). Bicyclic, tricyclic, and polycyclic (4+ rings) configurations were observed, with bicyclic by far the most common (Fig. 17A). There were 35 different bicyclic rings noted, of which those with two or three heteroatoms were the most common (34% and 37% respectively, Fig. 17B). Only four different atom types were seen in heterocyclic bicyclic rings. Nitrogen was the most common atom type, observed in 31 of the 35 bicyclic ring systems (Fig. 17C) and occurring 96 times in all when the presence of multiple nitrogen atoms per heterocycle was accounted for (Fig. 17D). Oxygen appeared in five bicyclic heterocycles (one of which, benzodioxole, contains two oxygens), sulfur in five, and boron once.

**Fig. 18:**
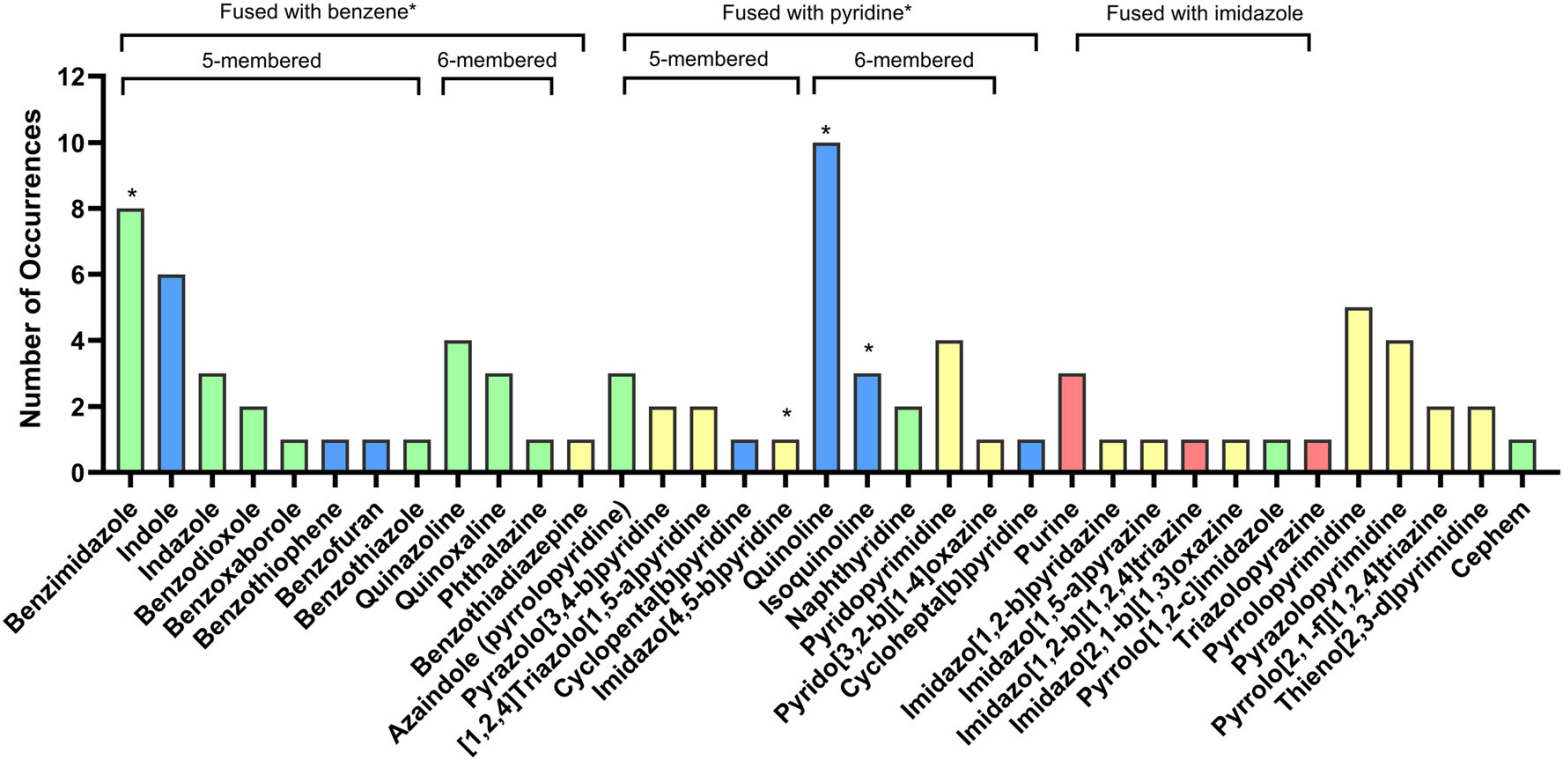
Overview of bicyclic heterocycles in EMA approved drugs (2014–2023). Colour key: blue: contains one heteroatom; green: contains two heteroatoms; yellow: contains three heteroatoms; red: contains four heteroatoms. *Heterocycles that fall into more than one category are: benzene fused with pyridine (quinoline and isoquinoline), benzene fused with imidazole (benzimidazole), and pyridine fused with imidazole (imidazo[4,5-*b*]pyridine).

As the degree of ring conjugation increases the complexity and diversity of the heterocycles also rises. As intricacy increases, common fragments amongst the fused configurations become rare. It becomes difficult to classify the more diverse and unique fused heterocycles as each one is seldom seen. Due to this, only the most common bicyclic configurations will be discussed. Interestingly, although no NAS contained an isothiazole ring, it was noted as part of a fused benzothiazole ring in the NAS lurasidone. Likewise, there were no NAS that contained an oxaborolane ring and the only time this ring was seen was when it was fused to a benzene ring in the NAS crisaborole (present in the now withdrawn dermatology ointment drug Staquis). Benzene, pyridine, imidazole, and pyrimidine were the most common rings observed as part of bicyclic combinations (Fig. 18).

The only 4-membered fused ring in the dataset was the 4-membered azetidine ring, fused with a thiazine, in the antibiotic ceftolozane (Fig. 3).

#### Heterocycles fused with a benzene ring

Heterocycles coupled with a benzene ring were the most diverse group of fused heterocycles seen in the EMA review. A benzene ring was seen together with many 5, 6 and 7-membered heterocycles. There were eight unique 5-membered heterocyces fused to a benzene ring. Benzimidazole, indole, indazole and benzodioxole were present more than once (Fig. 18).

#### Benzimidazole and indazole

**Fig. 19:**
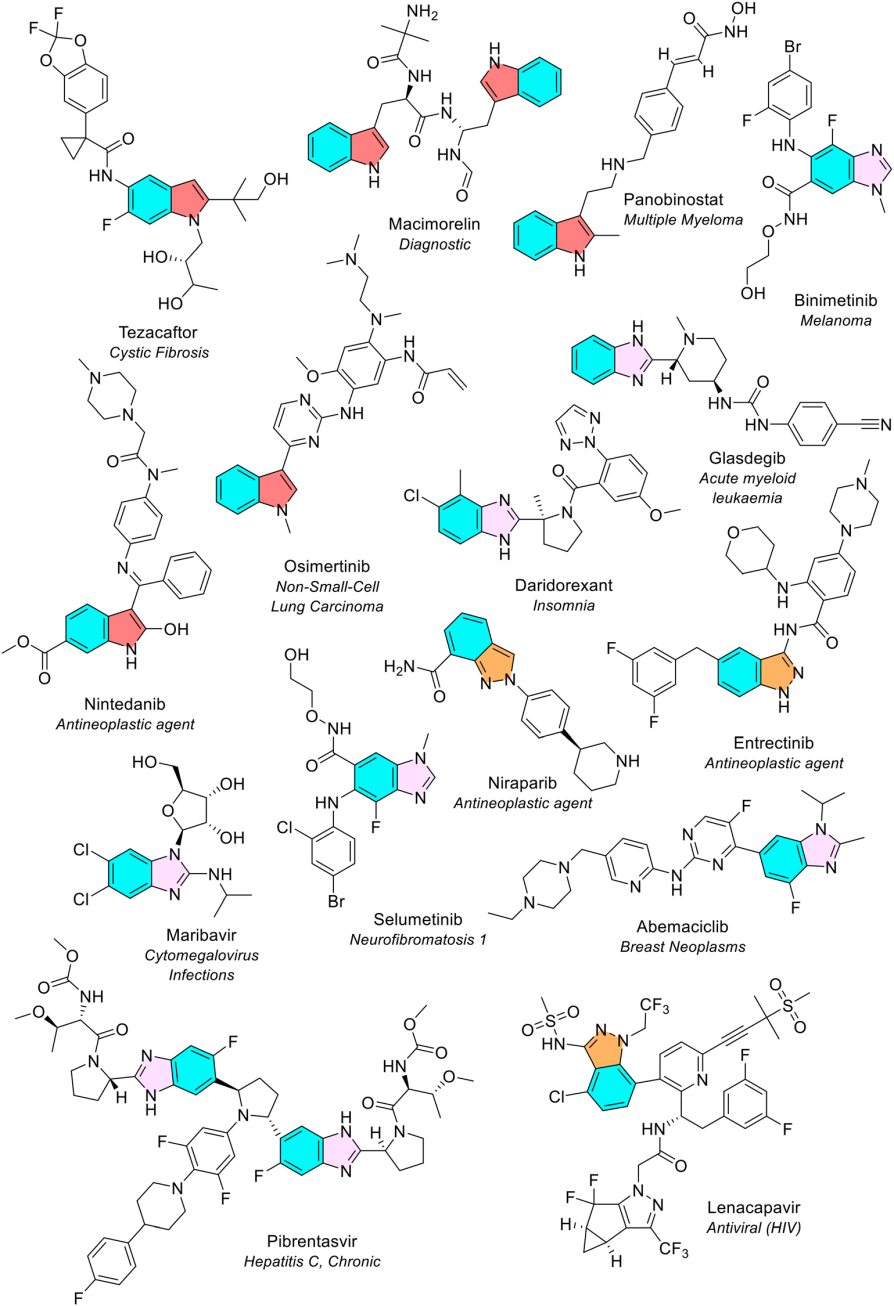
NAS approved by the EMA between 2014–2023 containing 5-membered heterocycles fused with benzene (part 1). Colour key: blue/orange: indazole (benzopyrazole); blue/red: indole (benzopyrrole); blue/pink: benzimidazole. *To avoid duplication, quinoline and isoquinoline-containing NAS are illustrated in*Fig. 22.

Benzimidazole and indazole are structural isomers. Benzimidazole was the most common 5-membered heterocycle fused with benzene, observed eight times in seven different NAS (blue/pink, Fig. 19). Both FDA reviews noted benzimidazole as the second most common 5-membered bicyclic heterocycle.^4,5^ Indazole (also known as benzopyrazole) was the third most common 5-membered heterocycle fused with benzene, noted three times in three different NAS (blue/orange, Fig. 19). The initial study of FDA approved drugs did not note the indazole ring.^4^ The updated review of FDA approved drugs between January 2013 – December 2023 reported indazole as the seventh most common 5-membered bicyclic heterocycle, seen five times.^5^

#### Indole

Indole (also known as benzopyrrole) was the second most common 5-membered heterocycle fused with benzene. It was present 6 times across 5 different NAS (blue/red, Fig. 19). The examination of the substitution on the indole rings revealed monosubstitution (2 times), disubstitution (2 times), trisubstitution (2 times) and tetrasubstitution (seen once). The indole rings in the NAS osimertinib (used to treat non-small cell lung cancer) and tezacaftor were substituted on the nitrogen in the indole ring. Tezacaftor is one of three NAS in the first triple combination therapy for the treatment of cystic fibrosis and works by increasing the number of CFTR proteins on the cell surface. The original review of FDA approved drugs found indole as the most common 5-membered bicyclic heterocycle, seen a total of 17 times.^4^ The later FDA review noted that indole was still the most common 5-membered bicyclic heterocycle occurring 21 times.^5^ The number of occurrences of indole in the recent FDA review is much higher than our data. The difference may be partially explained as the FDA review is inclusive of indole rings that are part of larger ring structures, for example in the NAS elbasvir the indole moiety is part of a larger 4-membered polycyclic ring (Fig. 26). Different medicines are also approved by each agency.

#### Benzodioxole

**Fig. 20:**
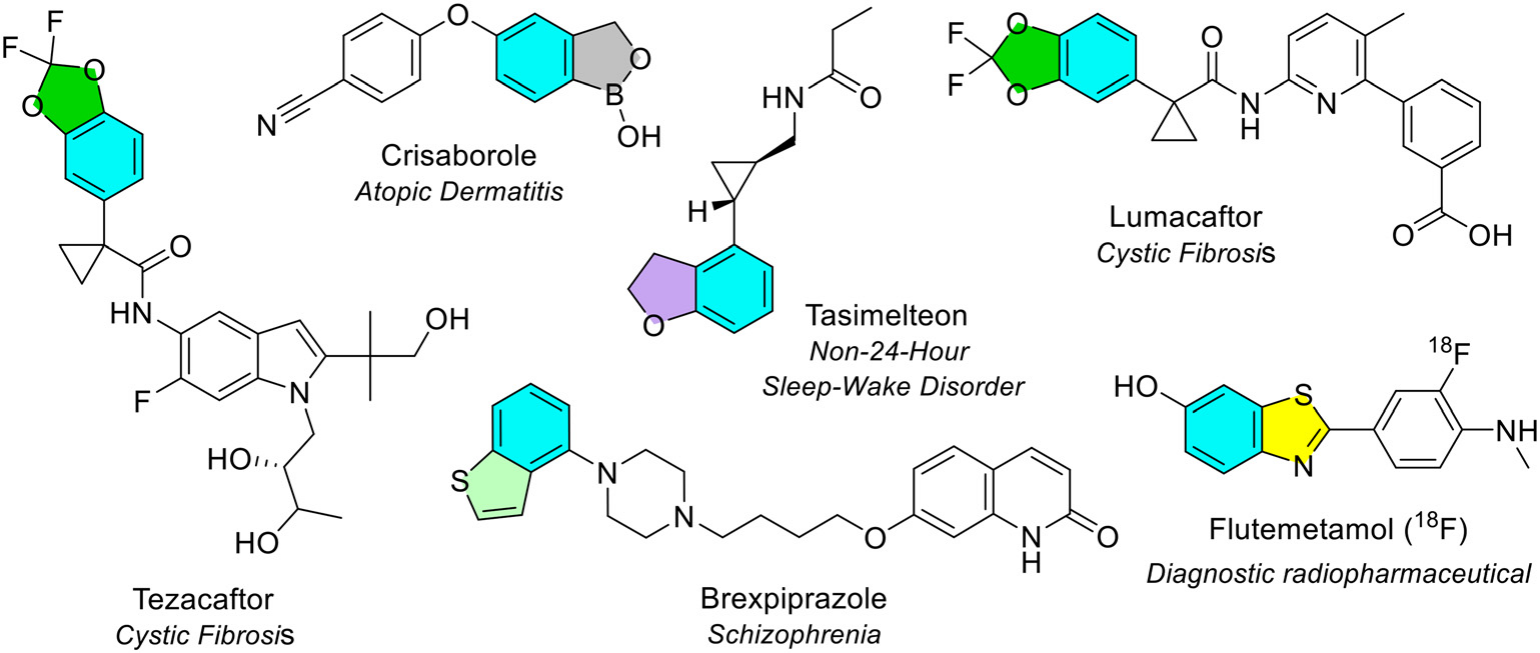
NAS approved by the EMA between 2014–2023 containing 5-membered heterocycles fused with benzene (part 2). Colour key: blue/green: benzodioxole; blue/light green: benzothiophene; blue/purple: benzofuran; blue/yellow: benzothiazole; blue/grey: benzoxaborole.

Benzodioxole (benzene fused with a dioxolane ring) was an uncommon 5-membered heterocycle fused with benzene. It was the only other 5-membered ring type to be seen more than once, observed twice in two different NAS (blue/green, Fig. 20).

#### 6- and 7-Membered heterocycles fused to a benzene ring

**Fig. 21:**
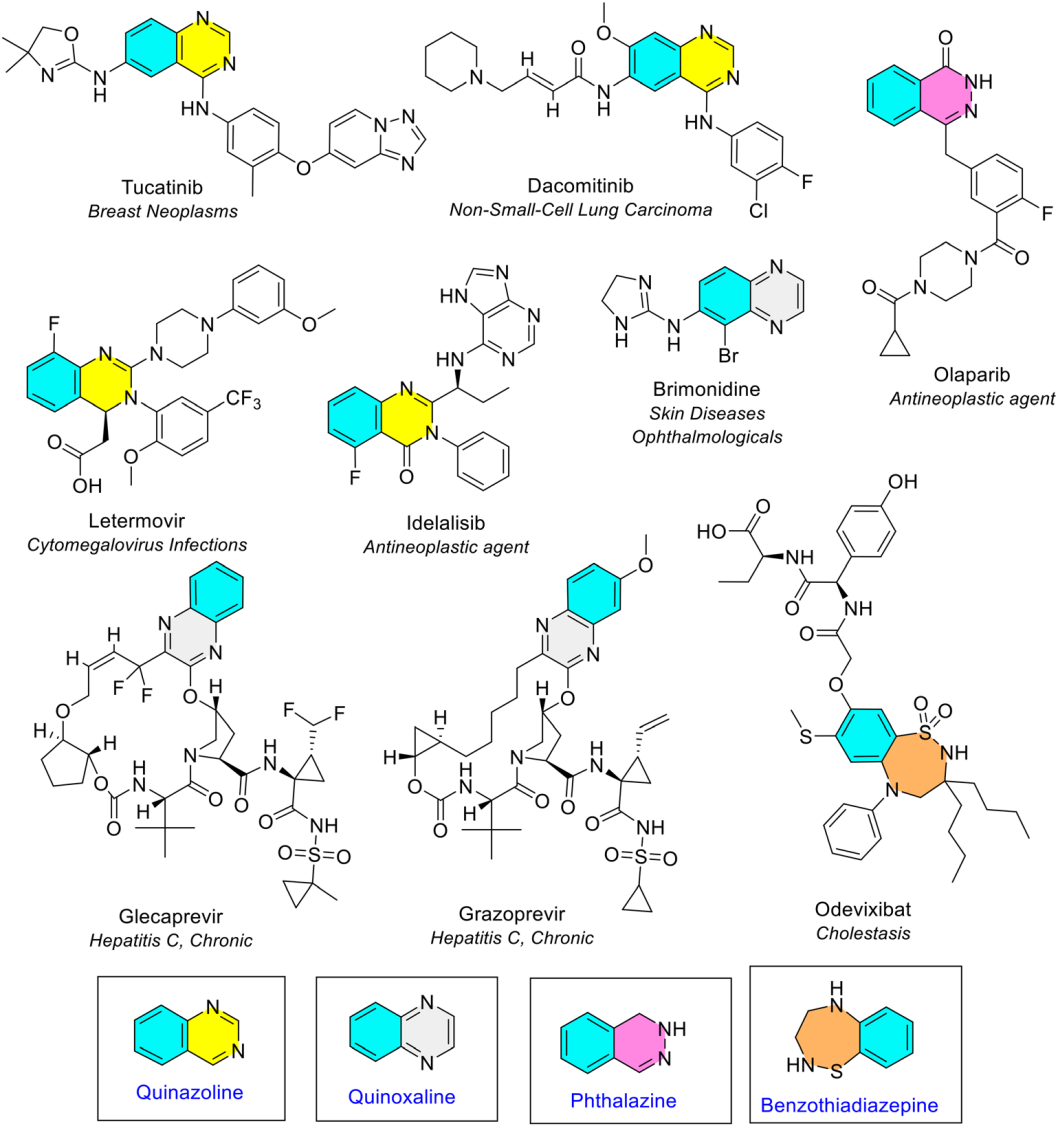
6- and 7-Membered heterocycles fused with benzene in NAS approved by the EMA (2014–2023); colour key: blue/yellow: quinazoline; blue/grey: quinoxaline; blue/pink: phthalazine; blue/orange: benzothiadiazepine.

Five unique 6-membered heterocycles (all containing nitrogen) and one 7-membered heterocycle (containing sulfur) fused to benzene were noted (inclusive of quinoline and isoquinonline, Fig. 18). Quinoline and isoquinoline are discussed in the next section (bicyclic rings containing pyridine). All three isomers of diazine were seen fused with benzene: quinazoline (benzopyrimidine), quinoxaline (benzopyrazine) and phthalazine (benzopyridazine) (Fig. 18). Quinazoline was the most common diazine heterocycle fused with benzene. It was seen four times in four different NAS (blue/yellow, Fig. 21). Quinoxaline was observed thrice in three different NAS (blue/grey, Fig. 21). In two instances, glecaprevir and grazoprevir, the quinoxaline was part of a larger ring system. Phthalazine was the final diazine heterocycle fused with benzene, and the least common. The only NAS that contained a phthalazine ring (in an oxidised form) was olaparib which is the NAS in the anti-cancer drug Lynparza (blue/pink, Fig. 21). There was only one 7-membered ring (thiadiazepane) fused with benzene, known as benzothiadiazepine. This was seen in odevixibat, the NAS in Bylvay, approved in 2021 as the first treatment for progressive familial intrahepatic cholestasis (PFIC) (Fig. 21).

#### Pyridine coupled with a second heterocycle

**Fig. 22:**
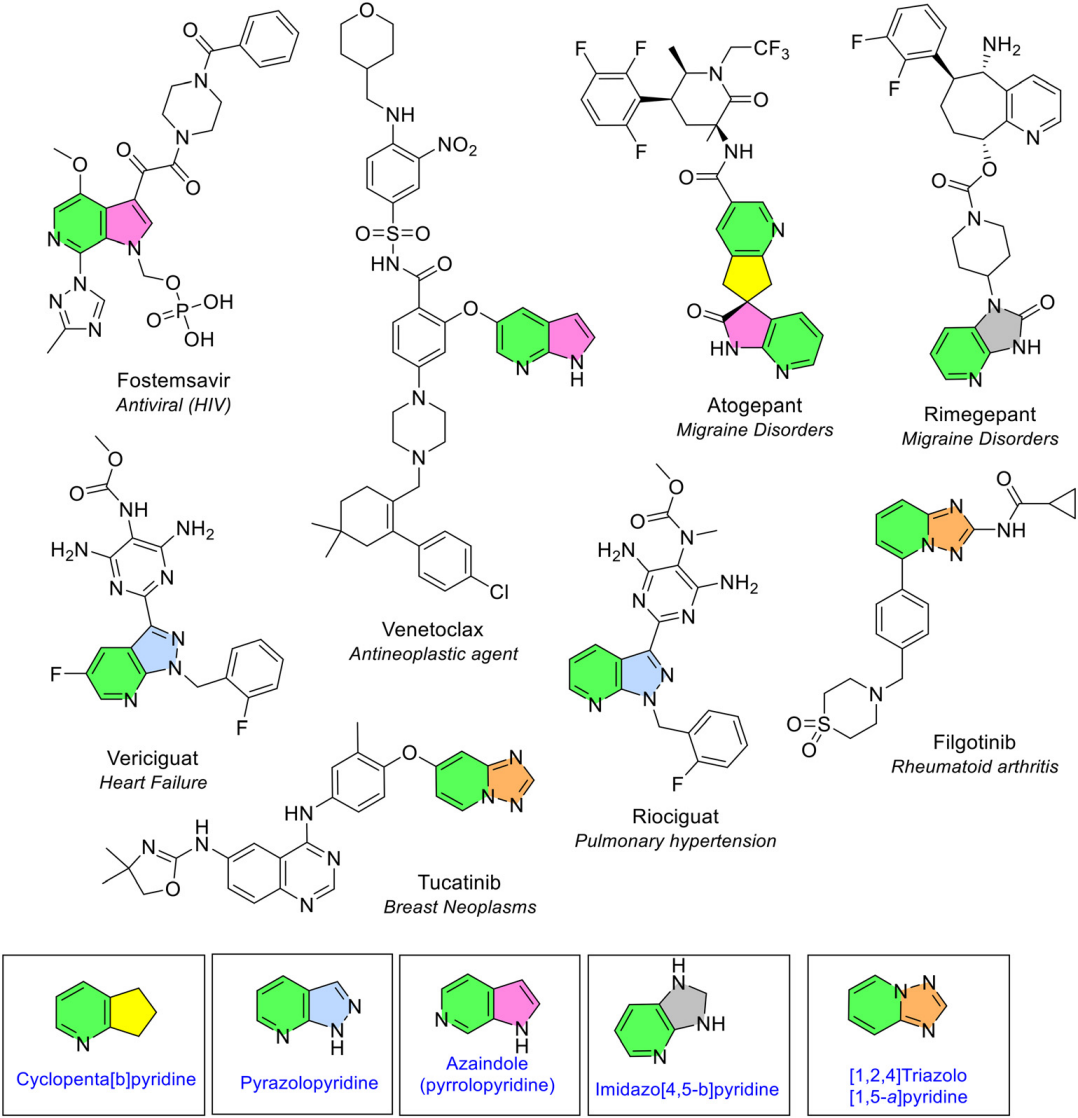
5-Membered heterocycles fused with pyridine in NAS approved by the EMA (2014–2023); colour key: green/pink: azaindole (pyrrolopyridine); green/blue: pyrazolopyridine; green/orange: [1,2,4]triazolo[1,5-*a*]pyridine; green/grey: imidazo[4,5-*b*]pyridine; green/yellow: cyclopenta[*b*]pyridine ring.

A pyridine ring was seen together with many 5, 6 and 7-membered heterocycles (Fig. 18). There were five different 5-membered heterocycles fused to pyridine (Fig. 22). Azaindole was noted three times in three NAS, and pyrazolopyridine and triazolopyridine were observed twice, each in two different NAS. Azaindole (pyrrolopyridine) consists of pyridine fused with a pyrrole ring (green/pink, Fig. 22). The NAS that contain the azaindole ring are fostemsavir (in the form pyrrolo[2,3-*c*]pyridine), venetoclax (pyrrolo[2,3-*b*]pyridine) and atogepant (also pyrrolo[2,3-*b*]pyridine). Atogepant contains a spiro arrangement of the pyrrolopyridine moiety with another bicyclic heterocycle, dihydrocyclopenta[*b*]pyridine, in a configuration known as 2-oxospiro[1*H*-pyrrolo[2,3-*b*]pyridine-3,6′-5,7-dihydrocyclopenta[*b*]pyridine]. The first review of FDA approved drugs did not observe the azaindole heterocycle, whilst the more recent review noted azaindole's presence six times (referred to as pyrrolopyridine).^5^

Pyrazolopyridine is pyridine fused with a pyrazole ring. The NAS that contain this ring type, vericiguat and riociguat, are structurally similar treatments for cardiovascular disease that contain the pyrazolo[3,4-*b*]pyridine ring (green/blue, Fig. 22). Triazolopyridine consists of pyridine fused with a triazole ring. Two NAS contain [1,2,4]triazolo[1,5-*a*]pyridine; tucatinib, used in treating breast cancer, and filgotinib which is used to manage rheumatoid arthritis (green/orange, Fig. 22).

#### 6- and 7-Membered heterocycles fused with a pyridine ring

**Fig. 23:**
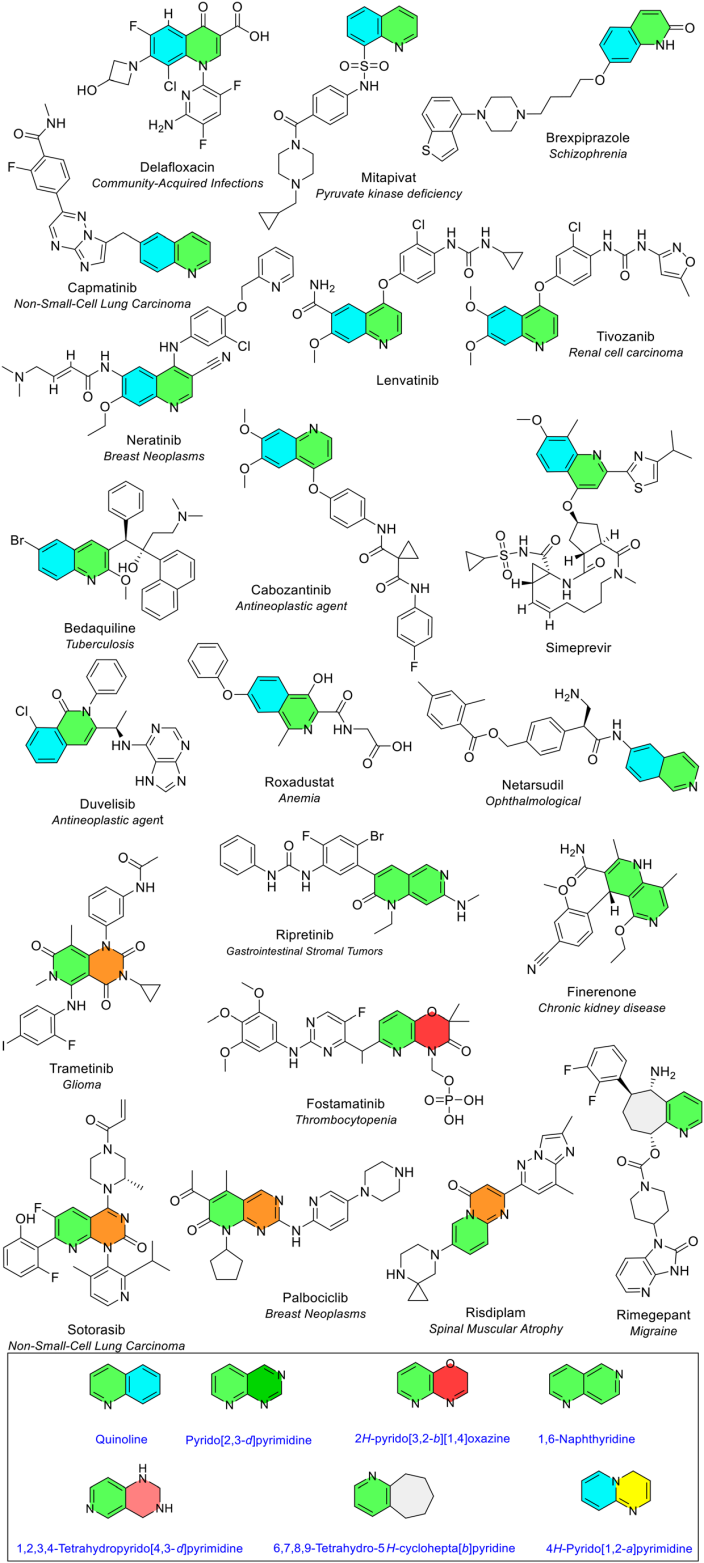
6 and 7-Membered heterocycles fused with pyridine in NAS approved by the EMA (2014–2023); colour key: green/blue: quinoline and isoquinoline; green/green: naphthyridine; green/orange: pyridopyrimidine; green/red: pyrido[3,2-*b*][1,4]oxazine; grey/green: cyclohepta[*b*]pyridine.

Six different 6-membered heterocycles and one 7-membered heterocycle fused to pyridine were observed. Two combinations were only seen once: pyrido[3,2-*b*][1,4]oxazine (fostamatinib) and cyclohepta[*b*]pyridine (rimegepant). The only NAS that contained a 7-membered ring fused with pyridine, in the form of a bicyclic cyclohepta[*b*]pyridine ring, was rimegepant. This is the NAS in the drug Vydura used to treat migraines, which is first-in-class antagonist of the calcitonin gene-related peptide receptor (CGRPR) (Fig. 23).

#### Quinoline and isoquinoline

Quinoline (1-quinoline) was the most common bicyclic heterocycle. It was seen ten times in ten different NAS (Fig. 18 and 22). There is significant interest in the quinoline ring due to its frequency in natural pharmacologically active compounds such as the alkaloid quinine. Extensive research on quinoline as a pharmacophore has also been conducted. This interest stems from quinoline's presence in drugs that treat a broad range of diseases and medical conditions.^21,22^ Isoquinoline (2-quinoline, a structural isomer of quinoline) was noted three times in three different NAS (Fig. 23). The recent FDA review notes quinoline's presence six times, whereas isoquinoline was reported three times.^5^

#### Naphthyridine

Naphthyridine appeared twice in two different NAS (green/green, Fig. 23). The naphthyridine ring belongs to a diverse family of fused rings known as diazanaphthalenes, two fused rings with the chemical formula C_8_H_6_N_2_. There are six possible positional isomers in the diazanaphthalene family, but both NAS, ripretinib and finerenone, contained the 1–6 naphthyridine isomer. Pyridopyrimidine was the only other bicycle containing pyridine plus another 6-membered ring (pyrimidine) to be seen more than once, observed four times in four different NAS in all instances as an oxidised form (green/orange, Fig. 23). The NAS that contain the pyridopyrimidine ring are sotorasib, in the form of a pyrido[2,3-*d*]pyrimidin-2-one ring, palbociclib in the form of a pyrido[2,3-*d*]pyrimidin-7-one ring and risdiplam in the form of a pyrido[1,2-*a*]pyrimidin-4-one ring (Fig. 23). Risdiplam was the first oral treatment for patients with certain types of 5q spinal muscular atrophy. Trametinib, a cancer medicine currently approved for melanoma and advanced non-small cell lung cancer, contains a pyridopyrimidine ring as the trioxidised trioxopyrido[4,3-*d*]pyrimidine ring.

#### Imidazole coupled with a second heterocycle

**Fig. 24:**
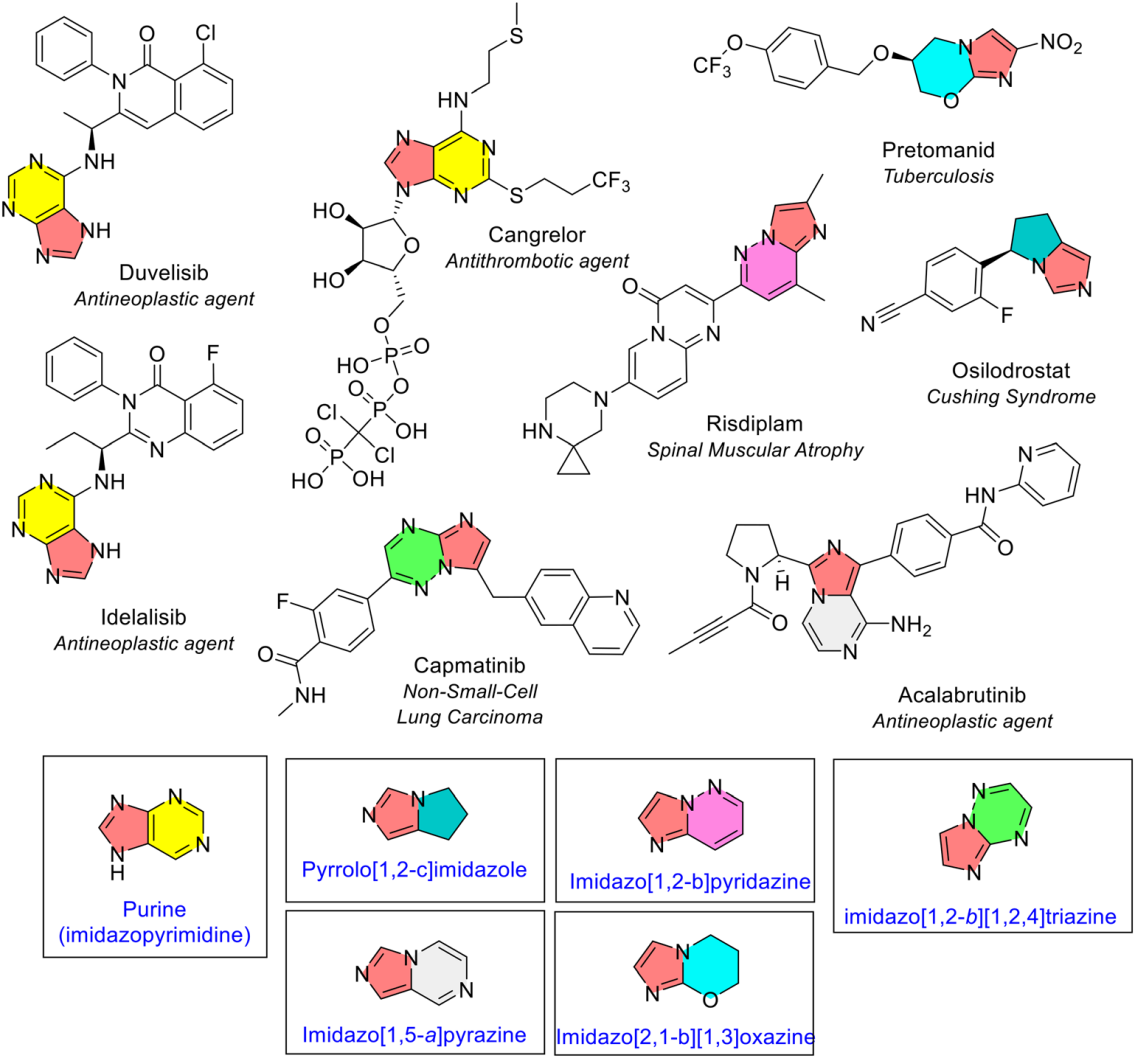
Imidazole fused with 5- and 6-membered rings in NAS approved by the EMA (2014–2023). Note that NAS containing benzimidazole and imidzao[4,5-*b*]pyridine are illustrated in previous figures; colour key: red/yellow: purine (imidazopyrimidine); turquoise/red: pyrrolo[1,2-*c*]imidazole; red/pink: imidazo[1,2-*b*]pyridazine; red/grey: imidazo[1,5-*a*]pyrazine; green/red: imidazo[1,2-*b*][1,2,4]triazine; blue/red: imidazo[2,1-*b*][1,3]oxazine.

Heterocycles that were fused with an imidazole ring were amongst the most common fused bicyclic combination, with nine unique combinations identified (Fig. 18 and 24). An imidazole ring was generally fused with 6-membered heterocycle, except for one combination with pyrrole (pyrrolo[1,2-*c*]imidazole) in osilodrostat. Most combinations were only seen once. Imidazole fused to benzene (benzimidazole, Fig. 19) and pyrimidine (purine, also known as imidazopyrimidine) were the only bicyclic systems noted more than once. Purine was present three times in three NAS (red/yellow, Fig. 24). Two of the three purine rings are monosubstituted in the pyrimidine part of the structure. Purine can also be found in many biopharmaceutical drugs, as it is a core structural fragment in the nucleotide bases adenine and guanine. Duvelisib is a cancer medicine used to treat chronic lymphocytic leukaemia (CLL), idelalisib is used to treat CLL in addition to follicular lymphoma, and cangrelor is an antiplatelet medicine used for reduction of thrombotic cardiovascular events in selected circumstances.

#### Pyrimidine paired with a second heterocycle

**Fig. 25:**
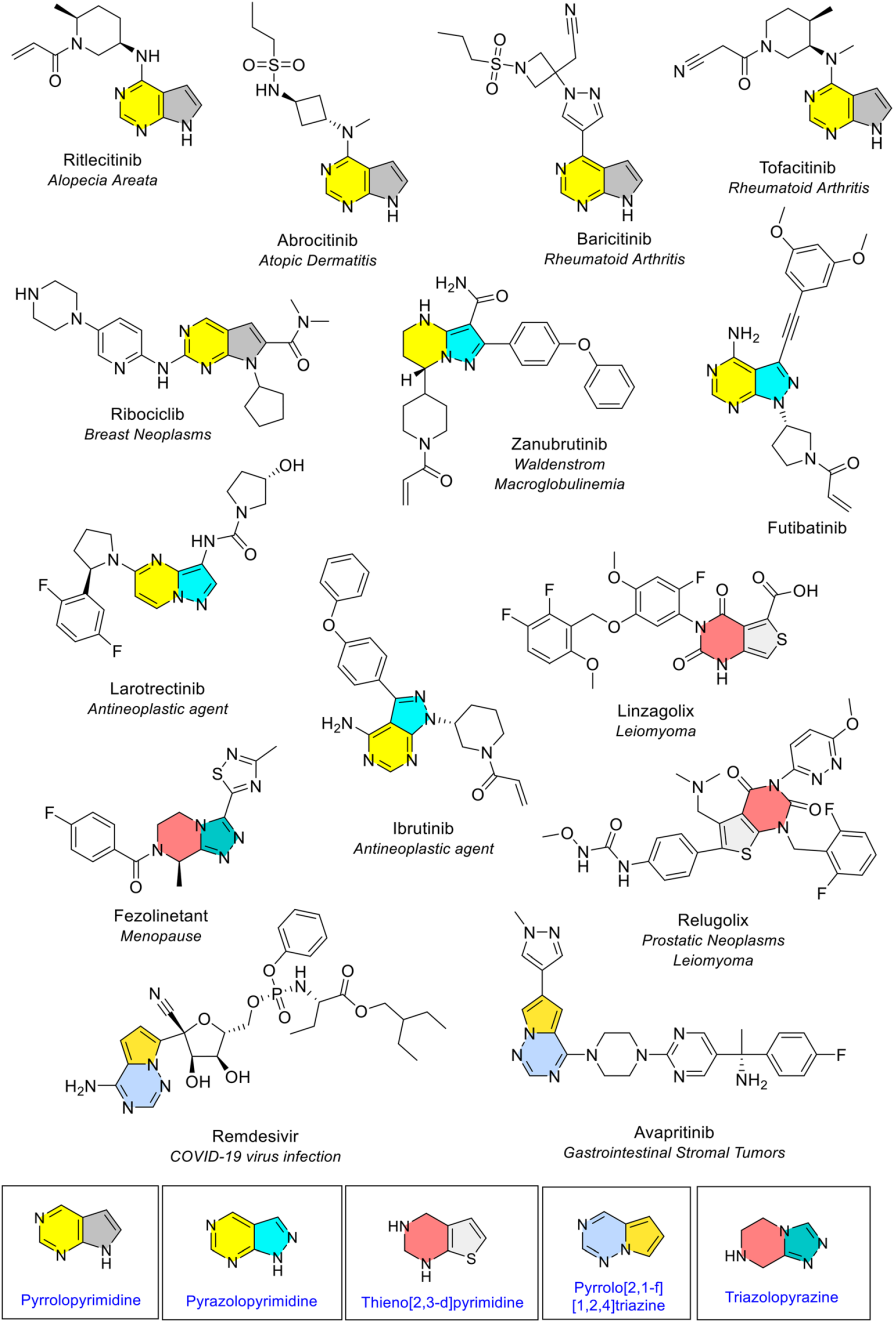
Further nitrogen and sulfur-containing bicyclic heterocycles in NAS approved by the EMA (2014–2023); colour key: yellow/grey: pyrrolopyrimidine; yellow/blue: pyrazolopyrimidine; red/blue: triazolopyrazine; red/grey: thieno[2,3-*d*]pyrimidine; blue/yellow: pyrrolo[2,1-*f*][1,2,4]triazine.

Heterocycles that were fused with or shared atoms with a pyrimidine ring were the final major grouping of fused bicyclic rings observed. The review noted four distinctive arrangements. Pyridopyrimidine (Fig. 23) and imidazopyrimidine (purine)(Fig. 24) were discussed previously. Pyrrolopyrimidine was the most common pyrimidine combination, noted five times in five different NAS (Fig. 25). As per the naming convention, its bicyclic arrangement is the 5-membered pyrrole ring fused with the pyrimidine ring. This arrangement may offer a “more diverse and potent pharmacological profile” than standalone pyrrole and pyridine rings.^23^ Pyrrolopyrimidine was reported to be present amongst many drugs that treat a wide range of diseases. This is supported by this EMA review as the NAS can be found in drugs used in dermatology, rheumatology and in cancer treatments.

Pyrazolopyrimidine consists of the 5-membered pyrazole ring fused with pyrimidine. It was seen four times in four different NAS, once each as the pyrazolo[1,5-*a*]pyrimidine form (zanubrutinib) and pyrazolo[1,5-*a*]pyrimidine form (larotrectinib), and twice as the pyrazolo[3,4-*d*]pyrimidine form (futibatinib and ibrutinib)(yellow/blue, Fig. 25). Futibatinib is a first-in-class fibroblast growth factor receptor (FGFR) covalent inhibitor for treatment of biliary tract cancer.

#### Further bicyclic arrangements

Several other bicyclic ring combinations were observed (Fig. 25). Two instances of oxidised thieno[2,3-*d*]pyrimidine rings were noted in relugolix and linzagolix choline. A pyrrolo[2,1-*f*][1,2,4]triazine structure was also noted twice. The NAS that contain this fused ring type are avapritinib which is used in treating cancer, and remdesivir which is used to treat and manage COVID-19 (Fig. 25). A triazolopyrazine ring was noted in fezolinetant in the form of triazolo[4,3-*a*]pyrazine. Fezolinetant is a first-in-class antagonist of neurokinin 3 receptors, approved in 2023 for treating hot flushes caused by menopause.

### Tricyclic and polycylic fused heterocycles

**Fig. 26:**
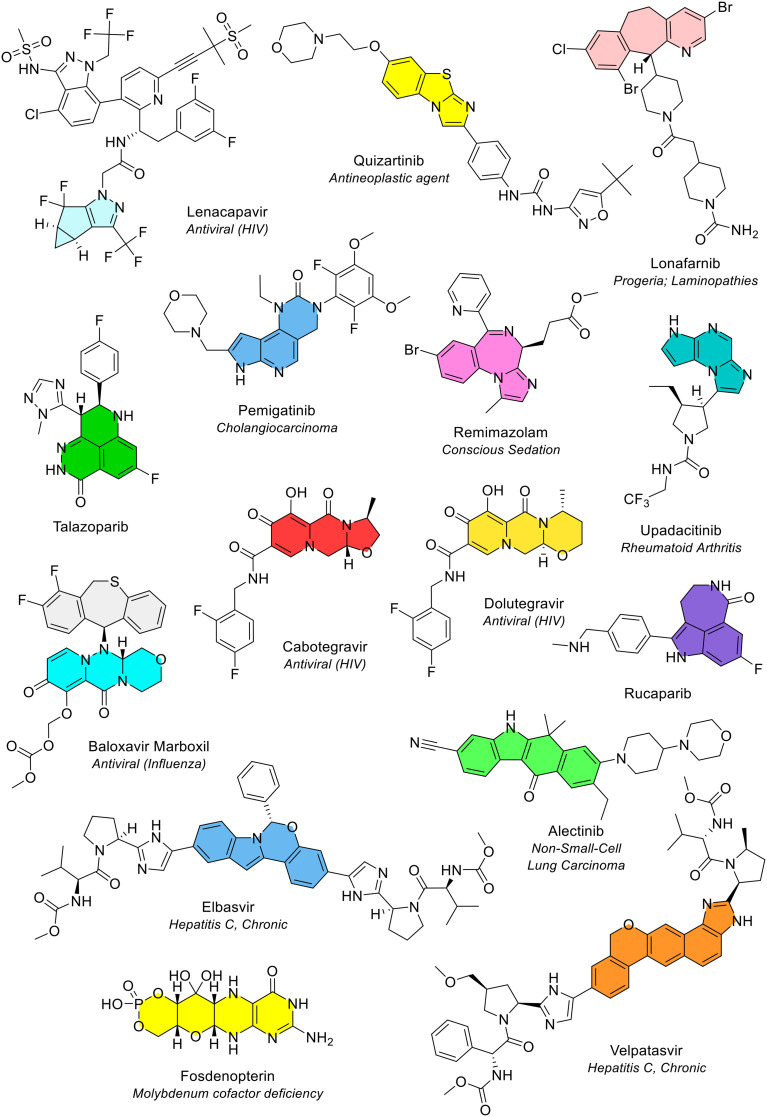
Tricyclic and polycyclic heterocyclic rings in NAS approved by the EMA (2014–2023).

This EMA drug approval analysis noted a tricyclic fused arrangement a total of 12 times across 11 different NAS (Fig. 26). A single tricyclic ring was seen in 10 separate NAS and one NAS, baloxavir marboxil, contained two tricyclic rings. Each tricyclic arrangement was only observed once. An arrangement consisting of four fused rings was seen three times in three different NAS, and one five-ring system was observed in velpatasvir (Fig. 26).

The review of the 164 NAS in the 10-year period observed four 7-membered heterocycles, all occurring in a fused configuration. One occurrence was as part of a bicyclic ring (the thiadiazepane ring in odevixibat, Fig. 21) and the other occurrences were part of tricyclic systems. The 7-membered heterocycles azepane and thiepane that contain a single heteroatom were seen once each. Azepane, a saturated ring that contains a single nitrogen atom, was present as part of a tricyclic ring (azepinoindole) in the NAS rucaparib (Fig. 26). The earlier FDA review found azepane as the second most common 7-membered heterocycle containing one heteroatom (seen three times, including in fused configurations).^4^ The more recent FDA analysis (2013–2023) listed azepane as the only 7-membered heterocycle containing one heteroatom (three times, including fused). A thiepane ring was observed once in a fused configuration where the thiepane ring is between two benzene rings (known as a benzo[*c*][1]benzothiepine ring), in baloxavir marboxil (Fig. 26). A 7-membered heterocycle containing two heteroatoms was observed once. This was the presence of a diazepine ring in a fused configuration in the NAS remimazolam, which contains the tricyclic imidazo[1,2-*a*][1,4]benzodiazepine ring (Fig. 26). This is the NAS in the medicine Byfavo, a sedative drug approved by the EMA for its use prior to surgery.

A dioxaphosphinane ring is a saturated 6-membered ring that contains two oxygen and one phosphorous atoms in the 1,2,3 position. It was seen in a fused configuration in fosdenopterin, the NAS in Nulibry (Fig. 26). This was the only phosphorus-containing heterocycle seen.

### Bridged, inorganic and spiro heterocycles amongst EMA approved drugs

**Fig. 27:**
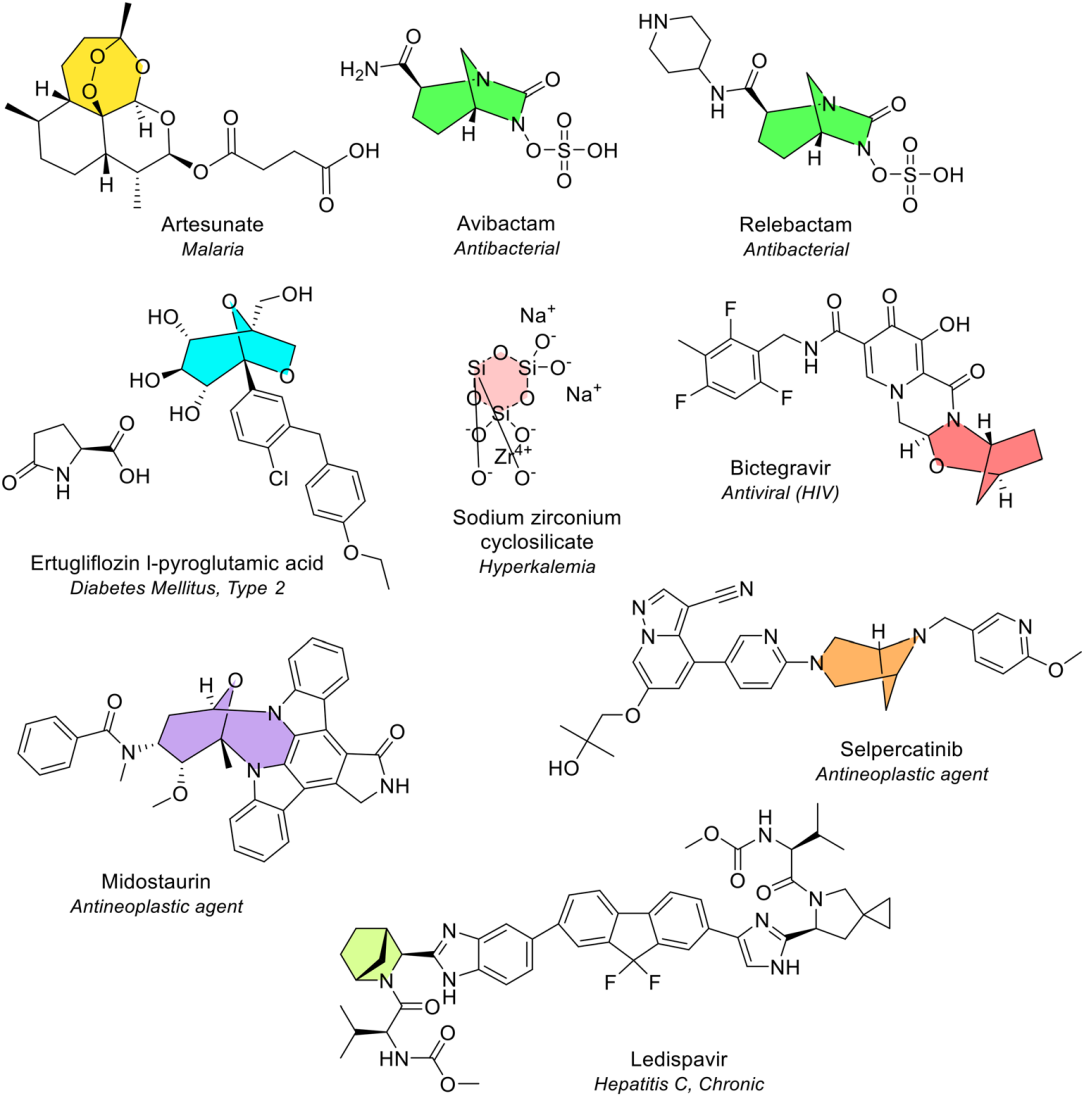
Bridged heterocycles and inorganic heterocycles in NAS approved by the EMA (2014–2023).

Of the 164 NAS, 5.5% contained either a bridged structure (eight NAS) or an inorganic heterocycle (one NAS) (Fig. 27). Bridged structures consist of two rings that share three or more atoms. The two tertiary carbon atoms connected through the bridge are termed bridgeheads and are separated by at least one other atom.

Of the eight NAS, only one bridged bicyclic heterocycle was seen more than once. Diazabicyclooctane is a bridged bicyclic saturated heterocycle that contains two nitrogen atoms and was present in two NAS. This ring type is also referred to as “DABCO” or triethylenediamine (TEDA). The NAS that contain the DABCO ring are avibactam and relebactam (Fig. 27). Both drugs are β-lactamase inhibitors used to treat complicated infections in combination with cepham-based β-lactam antibiotics.

Future efforts in drug design of bridged analogues will be influenced by the ‘escape from flatland’.^24^ Bridged compounds increase the three-dimensionality of drug candidates, which may decrease the risk of failure and improve binding selectivity and frequency. It will be of interest to track the emergence of bridged drugs over the coming years.

Of all 164 NAS, only one NAS contained an inorganic ring (Fig. 27). These types of heterocycles are unlike all other structures seen as they contain no carbon and are very rare in drugs. The inorganic ring, containing silicon and oxygen, was present in the NAS sodium zirconium cyclosilicate (Lokelma), used to treat hyperkalaemia.

**Fig. 28:**
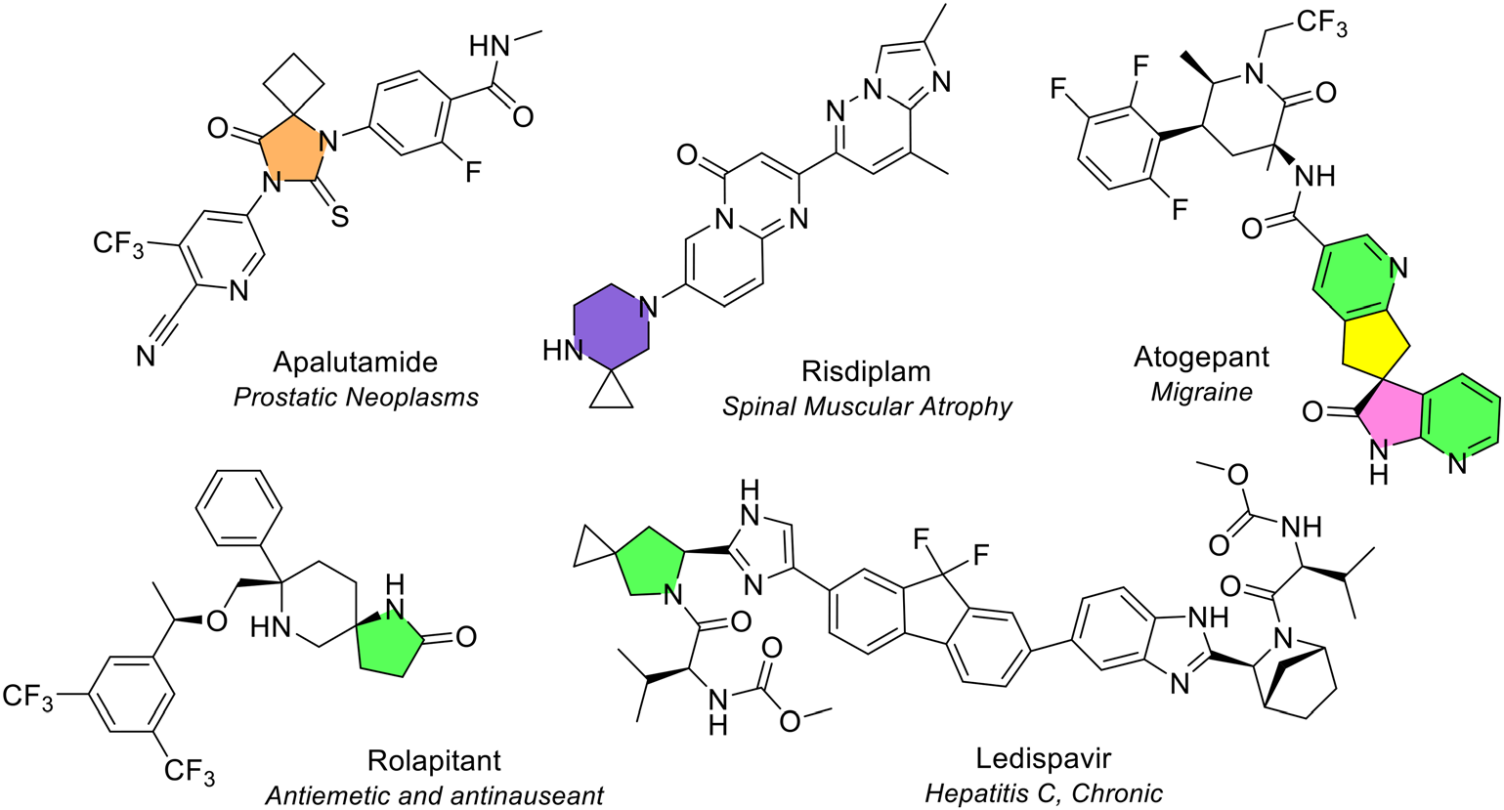
Spiro-configurations in NAS approved by the EMA (2014–2023).

As highlighted in previous sections, there were five NAS containing spiro-configured rings (at least one of which was a heterocycle) in the review period (Fig. 28). These five NAS were also approved by the FDA in the period 2013–2023. Other spiro-configured compounds approved by the FDA were ubrogepant, trilaciclib, sparsentan, lurbinectedin, and trabectedin. Of these, trabectedin was approved by the EMA in September 2007, sparsentan was approved in April 2024, and ubrogepant, lurbinectedin, and trilaciclib have not yet been approved by the EMA.

### Comparing heterocycles in FDA and EMA approved drugs

As mentioned, there have been two similar analyses conducted on FDA approved drugs. The first study published in 2014 detailed the structural diversity, substitution patterns and frequence of the nitrogen heterocycles amongst FDA approved drugs between 1938–2012.^4^ An updated extension to the initial work was carried out on FDA approved drugs between 2013–2023.^5^ Both excellent reviews contain detailed information about the structural diversity, substitution patterns, and frequency of nitrogen heterocycles. There were more heterocycles observed in the first FDA review (271 in total compared to 195, Table 1), attributable to differences in review periods (1938–2012 compared to 2013–2023). Four of the five most common heterocycles (pyridine, piperidine, pyrrolidine, and piperazine) were the same in both periods, with cepham falling out of the top 5 in recent times (Table 1).

**Table 1:** Top five most common heterocycles in EMA drug approvals (2014–2023) and FDA drug approvals (1938–2012 and 2013–2023)^a^

Order:	FDA approved	FDA approved	EMA approved
	(1938–2012):^4^	(2013–2023):^5^	(2014–2023):
1	Piperidine (72)	Pyridine (54)	Pyridine (29)
2	Pyridine (62)	Piperidine (40)	Piperidine (27)
3	Piperazine (59)	Pyrrolidine (40)	Pyrrolidine (26)
4	Cepham (41)	Piperazine (36)	Piperazine (20)
5	Pyrrolidine (37)	Pyrimidine (25)	Pyrimidine (18)

a Figures indicate the number of occurrences. FDA data classified several monocyclic rings and fused rings collectively. EMA data includes only rings occurring in monocyclic systems.

The five most common heterocycles are the same in the EMA and current FDA review (Table 1). There were slight differences in the way the heterocycles were classified between the EMA and FDA studies. For example, the FDA study did not always distinguish between monocyclic and fused heterocycle rings, whereas we classified fused rings separately. If fused rings were included, pyridine would still be the most popular heterocycle with 60 occurrences (29 monocyclic heterocycles plus 31 fused, Fig. 10 and 18). This underlines that there are a variety of classification approaches for analysing drug approval data. Consideration also needs to be given to classification of spiro configured NAS, which we have classified as separate rings while highlighting the nature of the spiro structures.

The EMA and recent FDA data is the same for the top five heterocycles, all containing nitrogen. Extending the analysis to the top ten from EMA data, there are popular non-nitrogen containing heterocycles appearing in drug scaffolds. For example, the eighth most common monocyclic heterocycle in EMA approved drugs was tetrahydropyran (oxane; 8 appearances) and tetrahydrofuran was the tenth most common amongst EMA approved drugs (oxolane; 7 times). Quinoline is the most popular fused heterocycle (10 occurrences) and would appear 7th on the overall list. As noted, FDA approvals have been analysed previously, but future work analysing drug approvals by other regulatory bodies would provide useful information concerning trends in other regions. The use of artificial intelligence to gather and analyse large datasets of regulatory information will undoubtedly be a trend in the coming years. Previous work has shown that synthesised chemical space is underutilised in drug discovery^25^ and the emergence of new heterocyclic scaffolds in drug discovery will be an interesting area to watch in the future.

## Conclusion

This was the first detailed analysis of the structural diversity of all heterocycle types in EMA drugs approved over a 10-year period (2014–2023). The initial search found 160 EMA-approved medicines containing 164 NAS with one or more heterocycles. Of 164 NAS, one heterocycle was seen in 24%, two in 36%, and three in 21%. The majority of the NAS contained a fused heterocycle (59%). The most common fused rings were bicyclic rings [quinoline (10 occurrences), benzimidazole (8), indole (6), pyrrolopyrimidine (5), quinazoline (4), pyridopyrimidine (4), and pyrazolopyrimidine (4)]. Tricyclic, polycyclic, bridged and inorganic rings were also present.

When examining the monocyclic heterocycles amongst the 164 NAS, there were 28 unique monocyclic heterocycles identified. The review found 3, 4, 5, and 6 membered standalone heterocycle rings across the NAS. Both saturated and unsaturated ring types were present. Nitrogen, oxygen, sulfur, and boron heteroatoms were observed. 5-Membered rings were the most diverse as they accounted for 15 heterocycles, the five most common being pyrrolidine (26 occurrences), pyrazole (14), triazole (8), imidazole (8), and tetrahydrofuran (7). 6-Membered rings were the second most diverse as there were 11 unique heterocycles, with the 5 most common being pyridine (29 occurrences), piperidine (27), piperazine (20), pyrimidine (18) and tetrahydropyran (8).

Historical influences are evident in drug structures, such as the continued use of beta-lactam based NAS for treatment of infection. The influence of endogenous ligand-based scaffolds, such as purine and pyrimidine, is also clear. Despite the large diversity in drug structures, several heterocycle types predominate. It will be interesting to see if such trends continue into the next decade and beyond, especially given potential impacts of artificial intelligences, green chemistry, and other emerging technologies on drug design.

## Author contributions

M. Ward: investigation, visualization, writing – original draft; N. M. O'Boyle: conceptualization, investigation, visualization, writing – review & editing, supervision.

## Conflicts of interest

The authors have no conflicts of interest to declare.

## Abbreviations

ATMP: Advanced Therapy Medicinal Products
CHMP: Committee for Medicinal Products for Human Use
DABCO: Diazabicyclooctane
EMA: European Medicines Agency
EPAR: European Public Assessment Report
EU: European Union
FDA: US Food and Drug Administration
HIV: Human immunodeficiency virus
IUPAC: International Union of Pure and Applied Chemistry
NAS: New active substance(s)
mAbs: Monoclonal antibodies
SPC: Summary of product characteristics
WHO: World Health Organisation

## Supplementary Material

MD-016-D5MD00403A-s001

MD-016-D5MD00403A-s002

## Data Availability

Supplementary information is available analysing the therapeutic areas of EMA approved new active substances (2014–2023) and the categories of new active substances approved by year. See DOI: https://doi.org/10.1039/D5MD00403A. The data supporting this article have been included as part of the SI. All data is also publicly available *via* the European Medicines Agency website.
