# Supplementary material for: Analysis of the structural diversity of heterocycles amongst European medicines agency approved pharmaceuticals (2014–2023)
Source: RSC Med Chem. 2025 Aug 11;16(10):4540–70. doi: 10.1039/d5md00403a (PMC12359206; doi:10.1039/d5md00403a)
Supplement: MD-016-D5MD00403A-s001 [file MD-016-D5MD00403A-s001.pdf]

Supplementary Information (SI) for RSC Medicinal Chemistry.
This journal is © The Royal Society of Chemistry 2025

***RSC Medicinal Chemistry 2025***

# Analysis of the Structural Diversity of Heterocycles amongst European Medicines Agency Approved Pharmaceuticals (2014–2023)

**Matthew Ward[1,2] and Niamh M. O'Boyle[1*]**

[1]School of Pharmacy and Pharmaceutical Sciences, Trinity Biomedical Sciences Institute, Trinity College Dublin, 152 - 160 Pearse St, Dublin 2, D02 R590 Ireland

[2]Viatris Damastown, Damastown Industrial Park, Damastown Road, Damastown, Dublin, D15 XD71 Ireland

## Supporting Information

## Table of Contents

**Figure S1.** Therapeutic areas of 380 EMA approved new active substances (NAS)(2014-2023) ........ 2

**Table S1.** Breakdown of EMA new active substance (NAS) approvals (2014-2023) by category and by year ........ 3

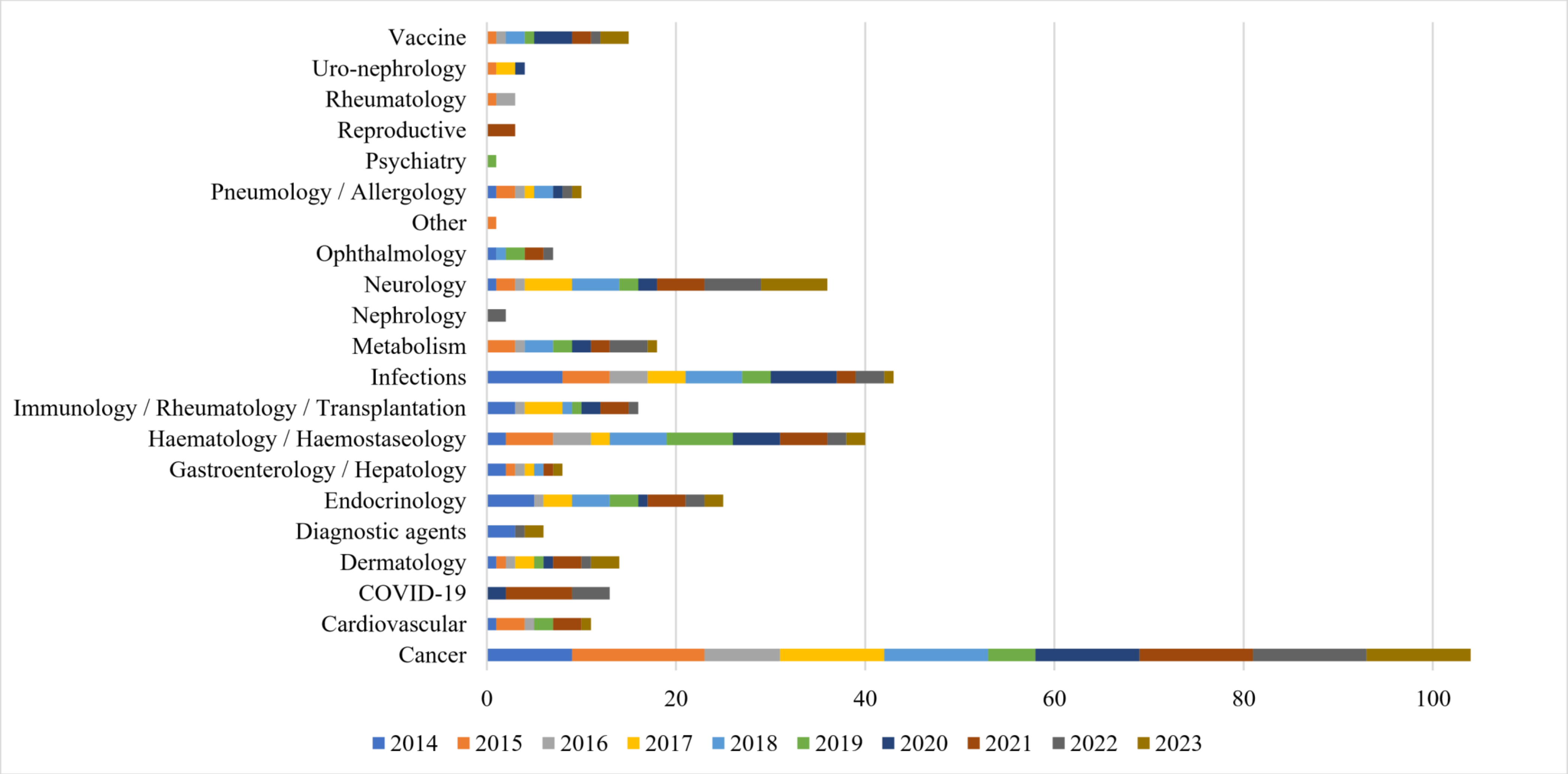


Figure S1. Therapeutic areas of 380 EMA approved new active substances (NAS)(2014-2023)

Table S1. Breakdown of EMA new active substance (NAS) approvals (2014-2023) by category and by year

| | Total | 2014 | 2015 | 2016 | 2017 | 2018 | 2019 | 2020 | 2021 | 2022 | 2023 |
|---|---|---|---|---|---|---|---|---|---|---|---|
| **Medicines containing a NAS** | 380 | 37 | 40 | 27 | 35 | 42 | 30 | 39 | 54 | 41 | 35 |
| **Medicines containing at least one NAS with a heterocycle** | **160** | **20** | **20** | **12** | **13** | **17** | **14** | **16** | **23** | **11** | **14** |
| **Medicines excluded *(*breakdown of categories below*)*** | 220 | 17 | 20 | 15 | 22 | 25 | 16 | 23 | 31 | 30 | 21 |
| **No heterocycle or heterocycle in an active substance previously approved** | 32 | 7 | 3 | 3 | 2 | 4 | 2 | 2 | 3 | 1 | 5 |
| **mAb** | 90 | 4 | 10 | 6 | 10 | 9 | 8 | 7 | 14 | 13 | 9 |
| **Peptide / glycoprotein** | 37 | 5 | 2 | 3 | 6 | 3 | 3 | 2 | 5 | 6 | 2 |
| **Cell therapy** | 13 | 0 | 0 | 2 | 1 | 2 | 0 | 2 | 2 | 3 | 1 |
| **Enzyme** | 10 | 1 | 2 | 0 | 1 | 2 | 1 | 1 | 1 | 0 | 1 |
| **Vaccine** | 22 | 0 | 1 | 1 | 1 | 2 | 1 | 5 | 5 | 3 | 3 |
| **Gene therapy** | 11 | 0 | 1 | 0 | 0 | 2 | 1 | 3 | 0 | 4 | 0 |
| **Antisense oligonucleotide** | 3 | 0 | 0 | 0 | 1 | 1 | 1 | 0 | 0 | 0 | 0 |
| **Triterpenoid** | 1 | 0 | 1 | 0 | 0 | 0 | 0 | 0 | 0 | 0 | 0 |
